# A Unified Treatment of Tribo-Components Degradation Using Thermodynamics Framework: A Review on Adhesive Wear

**DOI:** 10.3390/e23101329

**Published:** 2021-10-12

**Authors:** Lijesh Koottaparambil, M. M. Khonsari

**Affiliations:** Department of Mechanical Engineering and Industrial Engineering, Louisiana State University, 3283 Patrick Taylor Hall, Baton Rouge, LA 70803, USA; lijesh@lsu.edu

**Keywords:** adhesive wear, thermodynamic framework, steady-state wear, running-in, oscillatory operating condition, variable loading effects, variable speed effects

## Abstract

An extensive survey of open literature reveals the need for a unifying approach for characterizing the degradation of tribo-pairs. This paper focuses on recent efforts made towards developing unified relationships for adhesive-type wear under unlubricated conditions through a thermodynamic framework. It is shown that this framework can properly characterize many complex scenarios, such as degradation problems involving unidirectional, bidirectional (oscillatory and reciprocating motions), transient operating conditions (e.g., during the running-in period), and variable loading/speed sequencing.

## 1. Introduction

The performance of engineering systems largely depends on the interaction between the contacting surfaces in relative motion [1,2,3]. Irrespective of the types of interacting surfaces—i.e., solid, liquid, or gas—a common feature observed in every system is the effect of resistance to motion due to friction [3,4]. When one of the contacting surfaces is solid, the occurrence of friction is accompanied by the dissipation of energy and system degradation that causes wear [5,6,7], produces heat [8,9,10], and sound [11,12,13].

Wear is a progressive material loss from the contacting surfaces that reduces the useful life with a concomitant negative effect on performance and reliability. The deterioration of performance due to degradation accumulates until failure occurs [14,15]. This calls for the development of techniques to reliably predict the rate at which components degrade [16,17]. Therefore, it follows that the development of predictive wear equations capable of forecasting the useful life of tribo-components is vital for both the designer and the user of the engineering systems.

Many forms of wear equations have been proposed after Reye’s original hypothesis [18], but the relationship proposed by Archard [19] is the most widely accepted model. Commonly referred to as the Archard equation, it provides a satisfactory prediction for steady-state adhesive wear under dry operating conditions [20,21,22,23]. However, many recent investigations report that the quantitative predictions of the Archard equation for many processes—e.g., running-in [24], non-metals such as composites [25], situations that involve variable loading [26,27], and lubricated wear conditions [28,29]—tend to substantially deviate from measured values.

Having analyzed 300+ models and equations describing wear and friction from 5466 reports, Meng and Ludema [5] concluded that there is not a single or group of wear equations pertinent for general and practical use. Meng and Ludema [5] contended that the available predictive wear equations are confusing and that only a specialist can confidently employ them for the successful prediction of wear life. The challenge in developing equations arises due to the nonlinear, time-dependent, seemingly chaotic friction and wear behavior [2,3]. These observations point to the need for research in this area.

Fundamentally, the transformation of energy is always accompanied by the production of entropy during the degradation of a component. In a tribo-system, degradation is accompanied by material removal [30,31,32,33,34,35]. The same physics is involved when one deals with a deterioration of performance in batteries during charging and discharging [36,37], loss of consistency in grease due to shearing action [38,39,40,41], and accumulation of damage in cyclic fatigue [42,43,44,45]. Therefore, it is hypothesized that the degradation can be characterized using the framework of irreversible thermodynamics.

In this paper, we provide a detailed survey of attempts made at developing a unified wear equation for adhesive wear. Over a decade ago, Amiri and Khonsari [33] provided a review from the perspective of using the thermodynamic principles for contacting/sliding pairs. In the same year, Bryant [46] published a paper on the unification of different wear processes by considering the dissipative processes associated with sliding interfaces. While several researchers discussed the possibility of developing a unified wear equation using thermodynamic principles, until recently, sufficient experimental validation was lacking to demonstrate its efficacy in practice [47]. In what follows, we show that the recent advances in tribology have largely addressed these limitations by both in-depth theoretical and experimental investigations.

As a first step toward the development of unified wear equations, the present work focuses on characterizing the degradation of tribo-pairs due to adhesive wear under unlubricated conditions experiencing (i) different types of sliding motions that are either unidirectional or bidirectional; (ii) variable operating conditions in which either the load or the sliding speed or both change; and (iii) time-dependent or transient operating conditions, such as running-in.

The outline of the paper is as follows. In Section 2, a general introduction to adhesive wear and a review of the existing wear equations are presented. In Section 3, a detailed review of the wear equations for steady-state and running-in wear conditions is presented. This section comprises several subsections discussing the thermodynamic framework’s capability for characterizing adhesive wear in tribo-components that experience sliding motion in different operating conditions and directions. Furthera discussion on the capability of the thermodynamic principle in providing a unified wear equation for adhesive wear is carried out. Conclusions are presented in Section 4.

## 2. Adhesive Wear

**Figure 1:**
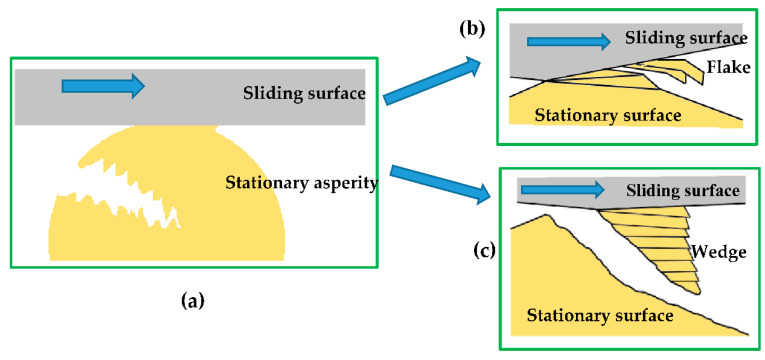
Schematic diagram representing the adhesive wear and types of wear particle formed: (**a**) breaking of asperity during sliding, (**b**) flake-like shear tongues particle and (**c**) wedge-like shape wear particle [63].

During adhesive wear [48,49,50,51,52,53,54,55,56,57], the asperities in the contacting interface tend to deform elastically and/or plastically under compression and shearing action [58,59,60,61]. The initiation of a crack in the plastically deformed asperities occurs through the combination of tensile and shearing fracture modes [62]. Once a crack is initiated, it propagates to the contacting interface and generates wear particles (see Figure 1a). The shape of the wear particles depends on the magnitude of the adhesive binding. Generally, the adhesive binding along the interface of asperities generates slip along the slip planes resulting in flake-like shear tongues (see Figure 1b). If the plastic deformation in the contact region is large, a wedge-like shape wear particle is formed (see Figure 1c) [63].

The adhesive wear mechanism was first investigated by Holm [64] in 1946 for electric contacts from the perception of the real contact area. The volume of material loss $w_{vol}$ is considered proportional to the applied load *P*, sliding distance *x,* and inversely proportional to the hardness *H* of the softer material. Accordingly, the expression for wear volume $w_{vol}$ is:

(1) $$w_{vol}=K\frac{Px}{H}\;$$

where K is a nondimensional proportionality constant called wear coefficient.

Holm interpreted wear as the removal of particles in the atomic level or layers at the junction. In 1954, Archard [19] and, later in 1980, Rabinowicz [65] attributed the wear mechanism as the removal of particles due to the fracture of the junction itself. The wear equation introduced by Archard [19] has the same form as Equation (1) and requires the determining of the wear coefficient by performing pin-on-disk measurements. Equation (2) shows the rate of increase in wear volume and is often referred to as the Archard equation or the Archard wear law. The parameter *K* is called the Archard wear coefficient. It is defined as “the probability that the asperities of the tribo- pair in sliding motion will deform plastically and wear out.” Archard equation has been extensively used to predict the wear of tribo pairs in the sliding contact [20,21,22,23,66,67,68].

(2) $${\dot{w}}_{vol}=K\frac{PV}{H}\;$$

where *V* is the sliding speed.

The values of *K* vary from 10^−15^ to 10^−1^, depending on the operating conditions and material properties. Welsh [69] and Vancoille [70] have stated that the value of *K* varies up to two orders of magnitude just by making small changes in the operating conditions. For example, for AISI 1045 carbon steel rings and pin, the wear coefficient values for mild wear were reported to vary between 4.1 × 10^−5^ to 1.9 × 10^−5^, and for severe wear, *K* is shown to vary between 3.5 × 10^−3^ and 5.4 × 10^−3^ [1]. Similarly, for wrought aluminum alloy (6061 AI), the *K* values are in the range of 1.4 × 10^−6^ to 6 × 10^−5^ for mild wear and 9 × 10^−5^ to 1.5 × 10^−4^ for severe wear. Such a drastic variation in *K* values makes it difficult to predict and validate experimental results. Researchers nowadays agree that a systematic study on the *K* values is needed to better understand and characterize the wear behavior of tribo-components [5].

**Figure 2:**
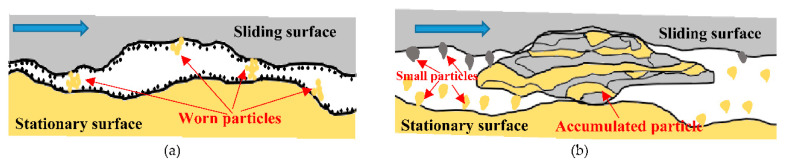
Type of wear: (**a**) mild wear, (**b**) severe wear [73].

As a result of the observations made by Holm [64] and Archard [19], researchers [71,72] have reported that the size of the particles detached from the contacting surfaces is dependent on the type of adhesive wear (i.e., mild or severe wear) and the geometry of the zone in compression and shearing [42]. For mild wear, the particle size is less than a few micrometers due to the presence of chemisorption activity (see Figure 2a). If the chemisorption activity is weak, the wear particles accumulate easily and quickly to form larger transfer particles that range from a few tens to a few hundreds of micrometers (see Figure 2b) [73].

The wear equations proposed by Archard [19] and Holm [64] cannot differentiate or explain the types of adhesive wear. Rather than deciding based on the probability of the removal of wear debris merely by the wear coefficient [74], the removal of wear particles from the contact surface must necessarily be dependent on the operating conditions, interfacial adhesion/friction conditions, and the physical, chemical, and mechanical properties of the contacting pairs [28,75,76,77].

Different approaches to characterize adhesive wear have been proposed that consider (i) physical and chemical process of wear [74], (ii) surface topography (fractal) parameters [78,79,80,81], including interfacial adhesion characteristics [82], and (iii) using irreversible thermodynamic principles [82,83,84,85,86,87,88,89]. The adhesive wear equations from different publications [64,73,74,79,81,82,83,87] are consolidated in Table A1 in the Appendix A. Referring to Table A1, it may be concluded that the wear equation developed using fractal parameters [64,73,74,79,81,87] are complicated, demanding the determination of several variables, including the surface profile and its parameters. These factors make it very difficult for the practitioners to implement for estimating adhesive wear.

On the other hand, energetic and entropy-based approaches provide wear models that directly correlate the degradation of a tribo-pair due to wear with frictional energy and entropy flow. The development of these models is based on the widely accepted hypothesis that the interaction of two bodies in sliding motion results in the dissipation of energy compared to the prevailing operating condition such as applied load and sliding speed.

Energy dissipation-based wear models are derived according to the first law of thermodynamics. Energy is dissipated through friction when two contacting bodies are subjected to a relative sliding motion. Entropy generation models are developed because the interaction of bodies in a sliding motion causes permanent and irreversible changes. These irreversible changes tend to cause disorder in the system and generate entropy per the second law of thermodynamics. The details of these concepts are presented in the following section.

### 2.1. Thermodynamic Approach

In general, irreversible changes are accompanied by the transformation of energy in the form of entropy generation, i.e., the energy dissipation through friction and temperature. Therefore, it is postulated that the complex wear behavior of a tribo-system could be satisfactorily characterized using the principles of irreversible thermodynamics. A detailed review of the energy-based and entropy-based approach for characterizing the wear behavior of tribo-pairs is presented in this section.

Wear models that use the thermodynamic approach can be classified into two categories: (i) energy dissipation and (ii) entropy generation. In the first approach, degradation is characterized using the energy dissipated due to the prevailing friction during the contact [89,90,91,92,93,94,95,96,97,98,99,100,101,102]. It is postulated that frictional energy degrades the contacting surface through plastic deformation, fracture, tribo-chemical reactions, etc. In the second approach, the degradation of a tribo-pair is considered to be the direct consequence of an irreversible thermodynamic process involving friction and temperature [85]. Here, entropy, a fundamental thermodynamic property employed for characterizing disorder, is utilized as a measure of degradation.

#### 2.1.1. Wear Model Based on Energy Dissipation

The correlation of the energy dissipation and wear volume for different tribo-pairs has been reviewed in references [5,89,90,91,92] for different sliding directions, loads, operating conditions (such as relative humidity), materials, etc. [5]. The intensity of the frictional energy dissipated in the contact region in relative sliding motion is measured using a quantity called specific power of friction, $Q_{F}$. This quantity was first introduced by Matveevsky [89] in 1965. $Q_{F}$ measures the rate of energy generated by friction in the contact zone as expressed in Equation (3):

(3) $$Q_{F}=\mu p_{c}V$$

where *μ* is the friction coefficient, and $p_{c}$ specific contact pressure. Friction power intensity is employed to characterize ball-on-disk and fretting tests experiencing unidirectional and bidirectional sliding operating conditions.

In 1995, Plint [90] postulated that the severity of the wearing process can be determined by estimating the energy dissipation per unit area. Plint [90] introduced a quantity called energy pulse, *EP*, expressed as a product of $Q_{F}$ and the total time of travel in contact *t* (see Equation (4)). This quantity is employed for characterizing wear of gear teeth and automotive engine valve trains.

(4) $$EP=2Q_{F}t=2\mu p_{c}Vt$$

Mohrbacher et al. [91] developed a model for a tribo-pair experiencing bidirectional sliding motion by summing the product of tangential force $F_{t}$ and displacement loop, calling it the cumulative dissipated energy, *E_d_* (see Equation (5)).

(5) $$E_{d}=\sum F_{t}x$$

Huq and Celis [92] employed Equation (5) to characterize the ball-on-disk unidirectional sliding experimental results. They defined the wear rate in terms of volumetric loss per unit dissipated energy. The expression is shown in Equation (6).

(6) $$E_{d}=\mu PVt$$

**Figure 3:**
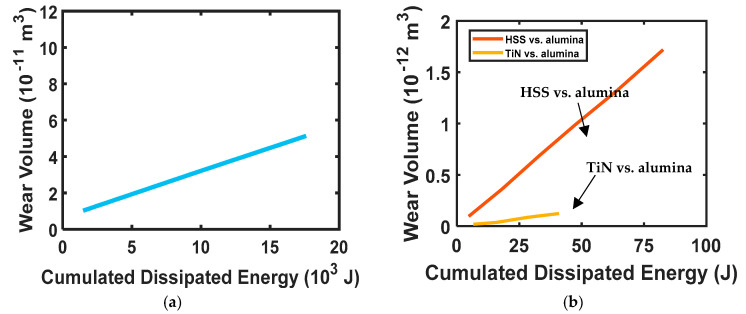
Linear fit between wear volume and cumulated dissipated energy (**a**) Huq and Celis [92] (**b**) Fouvry et al. [93].

To validate Equation (6), Huq and Celis [92] performed ball-on-disk experiments at the ambient condition of 50% relative humidity (RH) for different loads under a fixed sliding speed for TiN-alumina pair. They reported a linear fit of volumetric loss and energy dissipation (see Figure 3a) with an *R^2^* value of 0.96. The plot is obtained from the curve-fit equation $w_{vol}=0.64\times 10^{-11}+2.68\times 10^{-11}E_{d}$ provided in [92].

Fouvry et al. [93] investigated the wear behavior of TiN-alumina and high-speed steel (HSS)-alumina tribo-pairs by performing bidirectional sliding experiments and reported a linear correlation between wear volume and dissipated energy for both tribo-pairs with *R^2^* greater than 0.85. The linear fit is shown in Figure 3b. Similar observations were reported by Celis et al. [94] for hard-coated steel and alumina balls at different RH values.

**Figure 4:**
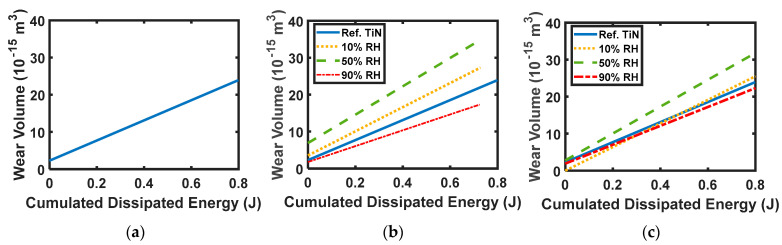
Linear fit obtained between wear volume and cumulated dissipated energy for different TiN coating tested against alumina, from Huq and Jean [95] (**a**) monolayer TiN coatings (**b**) monolayer (Ti,Al)N coatings, (**c**) multilayered 240 nm (Ti,Al)N:360 nm TiN coatings at different relative humidity (load 1 N, frequency: 10 Hz, linear displacement stroke of 100 μm).

Huq and Jean [95] attempted to establish a correlation between cumulated dissipated energy and wear volume by performing wear experiments on different types of coating and at various relative humidity conditions. For a monolayer TiN coating, a linear correlation for all RH conditions was observed (see Figure 4a). The linear fit for monolayer (Ti, Al)N coatings and multilayered 240 nm (Ti, Al)N:360 nm TiN coatings at 10%, 50%, and 90% relative humidity conditions are shown in Figure 4b,c, respectively. Both figures show that, unlike for monolayer TiN coating, the linear fit lines for different RH conditions are different. In other words, the correlation between the wear volume and energy dissipation is significantly affected by the surrounding operating conditions.

From an experimental study for different coatings, Fouvry and Kapsa [96] concluded that the Archard approach does not provide satisfactory results when the friction coefficient varies with time. Further, they established that characterizing the tribo-system through the dissipated energy approach would provide a stable quantification as the modeling approach integrates the friction coefficient in its formulation. The regression coefficient *R^2^* of experimental data for different tribo-pairs using the Archard equation was estimated to be varying between 0.64 to 0.86; while using energy dissipation, the *R^2^* value varied from 0.85 to 0.95.

From the correlation between the average wear rate and power dissipation, Aghdam and Khonsari [97] developed a property called a dissipation−wear rate factor to characterize the tribo-pair and their sliding configuration. They reported the existence of a linear correlation between the contact temperature and wear rate.

Indeed, the energy dissipation approach is promising. However, the characterization of the degradation process of the tribo-system becomes complex when the interaction of the surroundings is considered, and for such a tribo-system, a more improved approach is required [95].

#### 2.1.2. Wear Model Based on Entropy Generation

For a complex process like friction and wear, it is impractical to contemplate on pure mechanical phenomena without employing thermodynamics principles. These laws govern the energy flow processes and directly relate them to the nature of the contact [103]. Bikerman [104], in 1970, was the first to provide a short description stating the importance of studying the friction behavior of tribo-pairs from the thermodynamic perspective. Later, in the 1980s, Klamecki [105,106,107,108] provided a detailed thermodynamics analysis for the contacting bodies involving wear, friction, and entropy operating at nearly equilibrium conditions. In his first paper, Klamecki [105] attempted to clarify the wear process by considering entropy generation, energy, and mass conservation. Next, Klamecki [106] investigated the friction phenomenon by considering the energy transfer that occurred between two bodies and showed that frictional interaction between the contacting pair is a dissipative process. Further, he assessed the thermodynamic stability of entropy generation for small fluctuations about the nonequilibrium state. Subsequently, in another paper, Klamecki [107] evaluated the stability and entropy generation of different cases of the sliding condition through the thermodynamic framework. Employing the model proposed by Rigney and Hirth [109] and Heilmann and Rigney [110], Klamecki [108] suggested that for a sliding system, the energy input is dissipated by plastic deformation about the sliding interface, and entropy is generated. Klamecki’s papers [105,106,107,108] opened the door for characterizing the degradation of the tribo-system from the thermodynamic framework.

The thermodynamics of contacting bodies experiencing a third-body interaction was formulated by Zmitrowicz [103,111,112] by including the friction, wear, and heat generation phenomena. He used continuum mechanics, rational thermodynamics, and experimental results to develop the formulae and presented them in three parts [103,111,112]. In the first paper [103], a thermodynamical formulation for two bodies in contact with an interfacial layer or third body in between the contacting surfaces was developed using conservation equations for mass, momentum, angular momentum, energy, and entropy balance. The second paper [111] dealt with constitutive formulations for assessing the behavior of the sliding bodies and the interfacial layer or the third body. The formulation considered thermoelastic deformation and heat transfer in sliding contacts. In the third paper [112], Zmitrowicz derived constitutive equations for friction force, wear, and frictional heat within the thermomechanical framework. Klamecki’s [105,106,107,108] and Zmitrowicz’s [103,111,112] works concentrated on theoretical formulation without experimental validation. These papers did not provide a direct relation correlating the wear loss and entropy.

Abdel-Aal explored the correlation between the wear of the contacting surfaces to the thermal properties of the materials [101,113,114,115,116,117,118,119]. He concluded that the wear particles thermally dissipated the applied friction energy during the relative motion since contacting materials limit the rate of dissipation. Abdel-Aal found that the wear volume was dependent on the amount of energy dissipated. The energy dissipation depended on the contact temperature, and the transition in the wear mechanism depended on heat dissipation. He employed heat dissipation capacity (HDC) and the specific rate of heat dissipation (SRHD) to study the heat dissipation during the contact process. The HDC and SRHD plots exemplified the ability of the material to dissipate heat and the rate of heat dissipation of the applied thermal load, respectively. Abdel-Aal stated that these measures were related to entropy flow and entropy generation.

Further, in his work [118], Abdel-Aal defined a mechanically affected zone (MAZ), where heat transfers from higher temperature contact asperities to lower temperature sub-layers. He postulated that the wear behavior of the tribo-pair is significantly affected by the ability of MAZ to remove frictional heat away from the surface. Abdel-Aal’s work primarily concentrated on understanding the wearing process and its transition from the thermo-mechanical perspective.

Considering fretting wear as an irreversible thermodynamic process and transitioning near equilibrium, Dai et al. [120] reported that entropy reaches a maximum value and entropy generation concludes at equilibrium. They equated entropy flow to the entropy production and solved for wear by considering it as a mass flux component of entropy flow.

Doelling et al. [17] performed experiments on a tribo-pair operating in the boundary lubricated regime to establish the correlation between entropy flow and the degradation of the component due to wear. Using a calorimeter and Equation (7), entropy flow *S* was measured. In this equation Δ*Q*^(*n*)^ is the increment of heat input and *T*^(*n*)^ is the average surface temperature of the rider during the *n*th time interval. The equation correlating wear volume and entropy flow $S_{e}$ is provided in Equation (8).

(7) $$S_{e}{}^{\left(n\right)}=\overset{n}{\underset{}{\sum }}\frac{\Delta Q^{\left(n\right)}}{T^{\left(n\right)}}$$

(8) $$w_{vol}=K\frac{T}{\mu H}\;S_{e}$$

**Figure 5:**
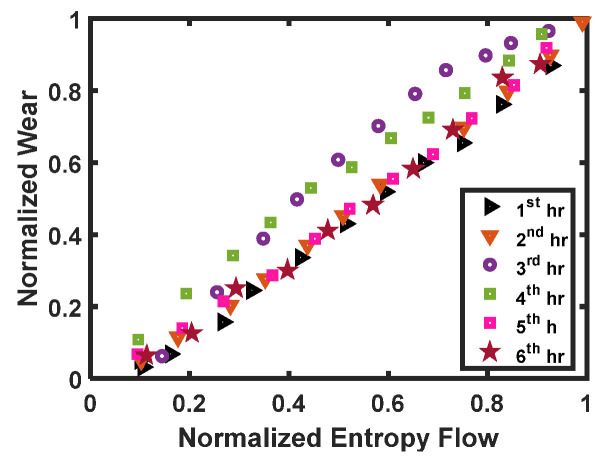
Normalized entropy flow vs. normalized wear at 9.1 kg load and 3.3 m/s [17]. Legends in the figure represents reading at different time of operation.

Doelling’s experimental results of normalized wear as a function of normalized entropy are shown in Figure 5. From this figure, a strong correlation between entropy flow and degradation of the component due to wear can be observed.

Doelling et al. [17] demonstrated that the Archard equation—that relates the wear rate to the applied load, sliding speed, and hardness pressure—is subsumed in their proposed wear−entropy relationship. It is worth mentioning that Doelling et al. [17] were the first to provide an explicit correlation between degradation and entropy generation for a tribo-system (see Equation (8)) with experimental validation.

In 2008, Bryant et al. [84] proposed a generalized theorem called degradation-entropy generation (DEG) to characterize the irreversible degradation of a steady-state wearing system under relative motion. This theorem relates the entropy generation to irreversible degradation via generalized thermodynamic forces *X* and degradation forces *Y*. The change in the entropy of the wearing open system—i.e., the system capable of exchanging both heat and mass with the surrounding area—is the sum of the entropy flow $d_{e}S$ and entropy generated $d_{g}S$ internally by the system [121] (see Equation (9)) and entropy generated is always positive (see Equation (10)).

(9) $$dS=d_{e}S+d_{g}S$$

(10) $$d_{g}S\geq 0$$

For steady-state wearing conditions, the change of entropy, *dS*, does not depend on time, i.e., *dS* = *0.* Therefore, it follows from Equation (9) that:

(11) $$d_{e}S=-d_{g}S<0$$

From Equation (11), it is inferred that for a steady-state wear condition, entropy flow and entropy generation are equal but have the opposite sign. This is a useful relationship since determining the entropy flow via experiments is more convenient than predicting entropy generation [17]. Nevertheless, it is the entropy generation quantity that defines the degradation of a tribo-system. Therefore, the concept of thermodynamic forces and flows needs to be carefully examined to derive appropriate formulas for the entropy generation in terms of experimentally measurable quantities [121].

Progress toward the objective of relating degradation and entropy generation is achieved by applying the DEG theorem reported by Bryant et al. [84]. Their derivation yielded a useful concept called degradation coefficient $B_{j}$ defined as the ratio of the thermodynamic degradation force(s), $Y_{j}^{k}$, divided by pertinent thermodynamic force(s), $X_{j}^{k}$. Equation (12) shows how this is applied to a system where the degradation force simply involves wear. Equation (13) shows a relationship between the rate of wear, ${\dot{w}}_{vol},$ to the degradation coefficient and entropy generation. The detailed steps followed to obtain the final equation are provided elsewhere [84] and for completeness of the paper, the derivation is reproduced in Appendix A.2.

(12) $$\;B_{j}=\frac{Y_{j}^{k}}{X_{j}^{k}}={\frac{\partial w_{vol}}{\partial_{g}S}|}_{p_{j}}$$

(13) $${\dot{w}}_{vol}{}_{j}=B_{j}{\dot{S}}_{g}{}_{j}$$

where ${\dot{S}}_{g}$ is the entropy generation rate, *p* is the degradation process consisting of *j* = 1, 2…*n* dissipative processes. These processes depend on time-dependent phenomenological variable $\zeta_{j}^{k}\left(t\right)$, *k* = 1, 2,…*m*.

The main features of the DEG theorem are summarized as follows.

- DEG theorem relates degradation to the irreversible dissipative processes to degradation mechanisms.
- The degradation coefficient measures how entropy generation and degradation interact [84].
- The theorem shows that for processes that involve multiple degradation mechanisms, ${\dot{w}}_{vol_{i}}$ will, accordingly, comprise of a corresponding entropy generation ${\dot{S}}_{i}{}_{j}$ and the associated degradation coefficient $B_{i}$.
- Equation (13) expresses the rate of degradation and entropy generation by applying the chain rule and does not assume the thermodynamic state of the system (Bryant [46]). Therefore, it can be applied to the systems operating far from equilibrium.
- The Archard law as well as the energy-based models for fretting wear are the corollary of the DEG theorem.

Now, assuming that the steady-state degradation of a tribo-pair is due to adhesion between contacting bodies in a relative sliding motion, in the absence of chemical reaction, the entropy generation can be determined from Equation (14).

(14) $${\dot{S}}_{g}=\frac{\mu PV}{T}$$

Substituting Equation (14) in Equation (13), yields

(15) $${\dot{w}}_{v}=B\frac{\mu PV}{T}\;$$

Now, comparing the wear equations Equations (2) and (15), a correlation between the degradation and the Archard coefficient can be established. The result is given in Equation (16).

(16) $$K=B\left(\frac{\mu }{T}\right)H$$

Bryant et al. [84] show that the Archard coefficient obtained from Equation (16) is in excellent agreement with published literature.

Examples provided in Bryant et al. [84] pertained to steady-state wear conditions. The applicability of the DEG for a transient wear condition like running-in condition was investigated by Lijesh et al. [86] in 2018. During the transient wear condition, the system is not in perfect equilibrium, and the change in internal energy *dU* and entropy *dS* is not zero [122].

Different sources of entropy may exist during the transient wear, such as (i) entropy generated at the interface *dS_g_*, (ii) entropy carried by the transfer of heat *dS_e_*, and (iii) entropy carried by the transfer of matters *dS_mt_*. By considering the first and second laws of thermodynamics, the entropy generation per unit control volume $\dot{\gamma }$ can be formulated using Equation (17) [86].

(17) $$\dot{\gamma }=\frac{\mu PV}{TA_{p}T_{hc}}-\frac{\dot{m}c\Delta T}{TA_{p}T_{hc}}+k\frac{{\left(grad\;T\right)}^{2}}{T^{2}}-\frac{\dot{m}}{A_{p}T_{hc}}\int_{T_{bulk}}^{T_{flash}}\frac{c}{T}dT$$

where $T_{hc}$ ≡ thickness of the control volume, $A_{p}$ ≡ area of the pin, $\dot{m}$ ≡ mass flow rate of wear particle, *c* ≡ specific heat capacity, *k* ≡ thermal conductivity, *T_flash_* ≡ flash temperature and *T_bulk_* ≡ bulk temperature. The terms on the right-hand side of Equation (17) represent the rate of frictional work done at contact temperature *T*, heat carried out by wear particle, the heat conducted, and entropy carried by the matters, respectively.

Lijesh et al. [86] established that the magnitude of the contribution of the second, third, and fourth terms in Equation (17) towards the entropy generation rate is diminutive compared to the first term. Further, for a steady-state wear regime, Aghdam and Khonsari [123] determined the ratio of the first and fourth terms is of the order of two. Therefore, the first term of Equation (17) that involves friction force dominates entropy generation. Now, the rate of entropy generation for the control volume is determined by:

(18) $$\dot{\gamma }=\frac{{\dot{S}}_{g}}{A_{p}T_{hc}}=\frac{\mu PV}{TA_{p}T_{hc}}$$

Comparing Equation (13) and Equation (18) yields degradation coefficient Equation (19), which is the same as Equation (15).

(19) $$\frac{{\dot{w}}_{vol}}{{\dot{S}}_{g}}=B=\frac{{\dot{w}}_{vol}T}{VP\mu }$$

Subsequently, during the running-in period, the wear rate changes with time (see Figure 4a). Thus, the degradation coefficient must be a function of time, i.e., *B* = *B_t_*(*t*), as represented in Equation (20). Suffix *t* represents the transient wear condition.

(20) $$\frac{{\dot{w}}_{vol}\left(t\right)}{{\dot{S}}_{g}\left(t\right)}=B_{t}\left(t\right)=\frac{{\dot{w}}_{vol}\left(t\right)T\left(t\right)}{VP\mu \left(t\right)}$$

The transient wear rate ${\dot{w}}_{vol}\left(t\right)$ for adhesive wear can be represented as provided in Equation (21). This equation is the rearrangement of an equation proposed by Pawlus [124].

(21) $${\dot{w}}_{vol}\left(t\right)={\dot{w}}_{s}\left[1+\left(\frac{{\dot{w}}_{0}}{{\dot{w}}_{s}}-1\right)e^{\left(-\tau_{w}t\right)}\right]$$

The change in friction coefficient and temperature equations [125] with time for transient and steady-state adhesive wear can be determined using Equations (22) and (23), respectively.

(22) $$\mu \left(t\right)=\mu_{s}\left\{1-\left[1-\frac{\mu_{0}}{\mu_{s}}\right]e^{\left(-t/\tau_{\mu }\right)}\right\}$$

(23) $$T\left(t\right)=\left[T_{0}+\frac{2Q}{\left(A_{p}{\left(\rho_{p}c_{p}K_{p}\right)}^{0.5}+A_{d}{\left(\rho_{d}c_{d}K_{d}\right)}^{0.5}\right)}{\left(\frac{t}{\pi }\right)}^{0.5}\right]\;$$

## 3. Efficacy of Degradation Coefficient *B*

### 3.1. Steady-State Wear

**Figure 6:**
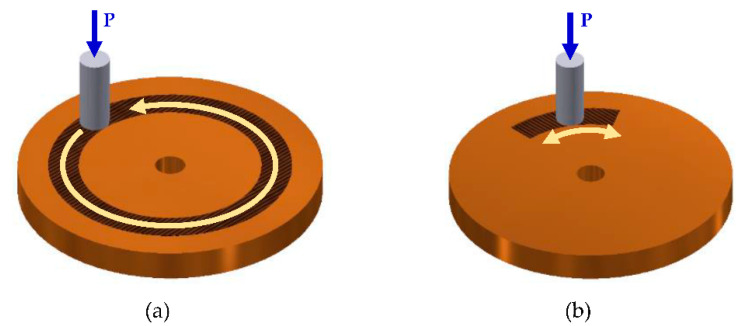
Different types of sliding rotation motion: (**a**) unidirectional and (**b**) bidirectional.

**Figure 7:**
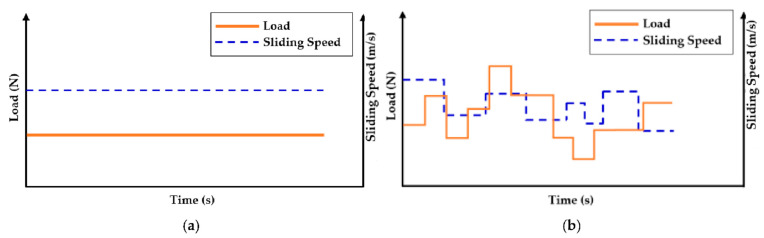
Different operating conditions: (**a**) uniform and (**b**) variable.

The efficacy of degradation coefficient in characterizing adhesive-type wear for a tribo-pair (i) sliding in uni- or bidirectional motions, (ii) operating in uniform or varying operating conditions, and (iii) transient wear conditions will be discussed in this section. Figure 6a,b display uni- and bidirectional motions in the rotating sliding condition. During variable operating conditions, the load or speed or both can change. Figure 7 shows an example of arbitrary varying loads and sliding speeds.

#### 3.1.1. Unidirectional Sliding and Uniform Operating Condition

Brahmeshwarkar [83], in 2006, developed an experimentally verified correlation between wear and entropy flow in a tribo-pair operating in a unidirectional sliding motion with uniform/fixed operating conditions. The materials chosen were bronze SAE 40 on steel 4140 and brass on steel 4140 tribo-pairs. The equation employed by Brahmeshwarkar [83] correlating the wear volume rate ${\dot{w}}_{v}$ and entropy flow rate (${\dot{S}}_{e}$) is the same as Equation (8) developed by Doelling et al. [17] for lubricated conditions. The value of entropy flow in Equation (8) was estimated by Equation (24)

(24) $$\dot{S}=\frac{A\frac{k_{s}\frac{T_{i}-T_{ii}}{d}}{1-\alpha }}{T}$$

where *A* is the contact area, $T_{i}$ and $T_{ii}$ are the temperatures measured by the thermocouples at two locations separated by distance *d* along the direction perpendicular to sliding, and α is the heat partitioning factor.

(25) $$\alpha =\frac{{\left(C_{p2}k_{2}\rho_{2}\right)}^{0.5}}{{\left(C_{p2}k_{2}\rho_{2}\right)}^{0.5}+{\left(C_{p1}k_{1}\rho_{1}\right)}^{0.5}}$$

where *C_p_*, k, and *ρ* are the specific heat capacity at constant pressure, thermal conductivity, and density of the materials 1 and 2, respectively.

**Figure 8:**
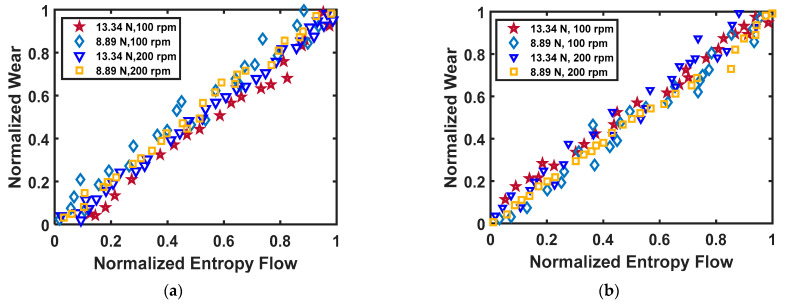
Normalized wear vs. normalized entropy flow: (**a**) for bronze SAE 40 on steel 4140, (**b**) brass on steel 4140 [83].

To validate Equation (8), Brahmeshwarkar [83] experimented with a tribometer by considering bronze SAE 40 on steel 4140 and brass on steel 4140 as tribo-pairs and the values of wear and entropy were determined. These values were normalized with respect to their maximum values, plotted in Figure 8. This figure shows that the normalized values of entropy flow and wear are proportional to each other.

In 2008, Bryant and Khonsari [126] provided correlations for dry sliding wear and entropy flow through a degradation coefficient. They determined the degradation coefficient (via Equation (15)) using the friction coefficient, temperature, and wear rate and obtained a wear coefficient using Equation (16). The slope of the wear−entropy results is precisely the degradation coefficient, *B*, as predicted by the DEG theorem.

**Figure 9:**
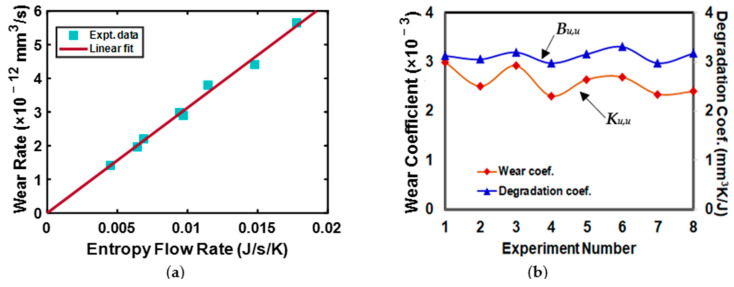
The experimental data and linear fit of power and entropy with respect to wear rate is plotted along with the comparison of the wear and degradation coefficients for the different experimental data provided by [123]. (**a**) Wear rate vs. entropy flow rate, (**b**) comparison of wear coefficient and degradation coefficient.

Considering the hardened steel 4140 and brass 360 tribo-pair in a ring-on-ring configuration under dry adhesive wear sliding conditions, Aghdam and Khonsari [123] performed an experimental validation of the thermodynamic principle. For a specific sliding regime, they observed that the degradation of the tribo-pair measured by wear rate was proportional to the power and entropy generation. Thus, they also concluded that the wear of the sliding system could be correlated to entropy generation through the degradation coefficient. To provide a clear understanding of their observation, the experimental data [95] along with the linear fit values for entropy generation for a series of measured wear rate values are plotted in Figure 9a. From this figure, it can be inferred that there exists an excellent linear correlation between the wear rate and entropy generation. Aghdam and Khonsari [123] concluded that irreversible thermodynamics is a promising tool for characterizing the wearing of tribo-pairs.

What follows next is that for different experimental data provided in Aghdam and Khonsari [123], the values of the Archard wear coefficient *K_u,u_* and the degradation coefficient *B_u,u_* are determined using Equation (26a,b) and plotted in Figure 9b. The suffix “*u,u*” represents unidirectional sliding with uniform operating conditions. From this figure, it can be inferred that *B_u,u_* has a lower variation for different operating conditions than the values of *K_u,u_*.

(26a) $$B_{u,f}=\frac{{\dot{w}}_{vol}T}{VP\mu }\;$$

(26b) $$K_{u,f}=\frac{{\dot{w}}_{vol}H}{PV}$$

Amiri et al. [127] in 2012 demonstrated that the formulae for the degradation coefficient *B* can be obtained by applying the Buckingham Pi theorem to derive a dimensional wear equation in terms of entropy. By performing a series of experiments on brass-steel and bronze-steel tribo-pairs, they established that the nondimensional groups comprising sliding speed, load, interfacial temperature, and the friction coefficient directly affect the heat generation rate. Hence, these parameters influence the entropy generated during the sliding of contacting bodies.

**Figure 10:**
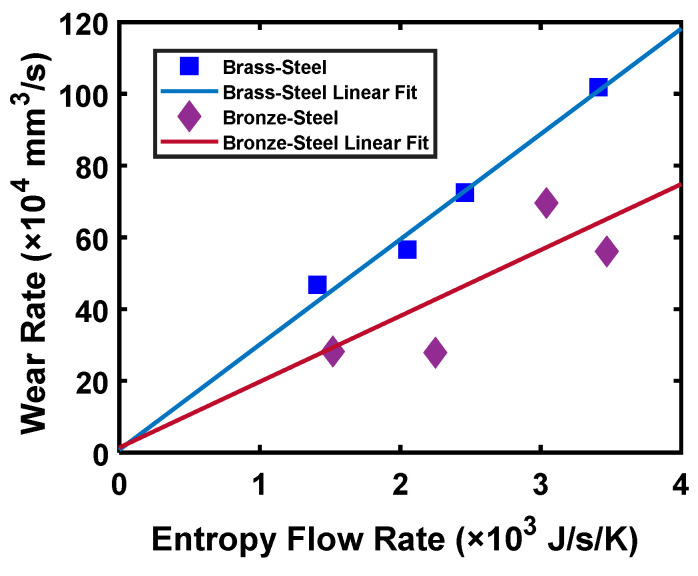
Wear rate vs. entropy flow rate for brass-steel and brass-steel tribo-pairs [127].

Using the experimental data from [127], the authors plotted the values of wear rate and the rate of entropy generation for brass-steel and bronze-steel tribo-pairs. The results are shown in Figure 10. From this figure, it can be observed that the values of the rate of entropy are proportional to the wear rate.

**Figure 11:**
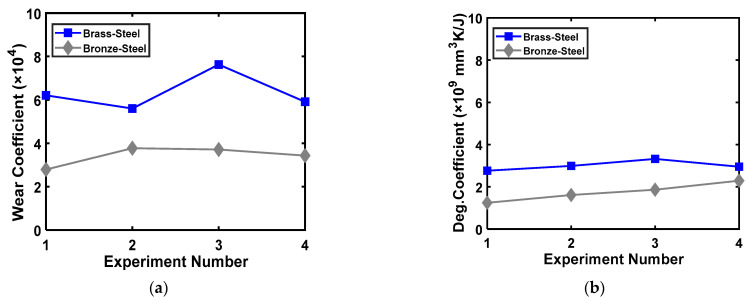
Comparison of wear and degradation coefficients for brass-steel and bronze-steel tribo-pairs for (**a**) brass−steel, (**b**) bronze-steel [127].

The values of coefficients *K_u,u_* and *B_u,u_* in the unidirectional sliding condition for brass-steel and bronze-steel tribo-pairs are shown in Figure 11a,b, respectively. Importantly, it can be inferred that the variation in the values of *B_u,u_* for the different experimental conditions are comparatively lower than *K_u,u_*. Lower variations of the coefficients in different experimental conditions imply that the coefficients are independent of experimental conditions. Therefore, this suggests that the *B_u,u_* determined using the DEG theorem can characterize material degradation better than *K_u,u_*.

#### 3.1.2. Unidirectional Sliding and Variable Operating Conditions

Numerous engineering components in the industry are subjected to varying loads and/or operating speeds. These components often tend to degrade faster than uniform operating conditions [128,129,130,131,132]. For example, according to Al-Tubi et al. [128], a gearbox used in wind-power experiences an 18.5% variation in torque and 13.5% variation in speed. These factors lead to micro-pitting in the gears. Turbulent wind fluctuations in the wind turbines result in a complex loading condition in the drive train [133]. Such variations in the operating conditions are responsible for significant damage and shorten the service life of bearings and gears [134,135,136]. Another documented example pertains to rollers used in sugar mills. The speed variation from 4 and 7 rpm is reported to have led to considerable deterioration in the bearing performance [137].

##### Loading-Sequence Effect

The discussions of the results in Section 3.1.1 were based on the reported results in references [78,79,95,98] in which the operating conditions were uniform. In this section, the efficacy of the DEG theorem is extended for characterizing the degradation of the tribo-pair during variable loading conditions.

**Figure 12:**
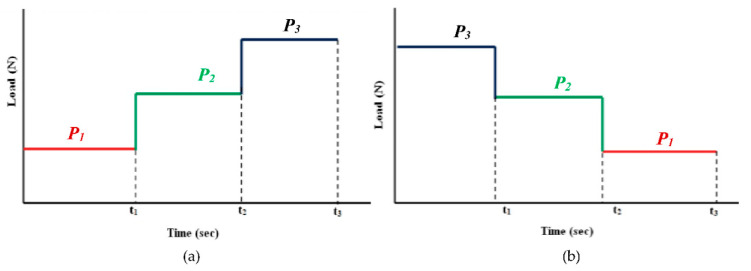
Loading sequences, (**a**) increasing load, (**b**) decreasing loads.

In 2018, Akbarzadeh and Khonsari [27] characterized the wear behavior of a tribo-pair under variable operating conditions under an increasing (from low-to-high) or a decreasing (from high-to-low) loading sequence by considering the Miner’s rule. See Figure 12a,b. In this Figure, *P*_1_, *P*_2_, and *P*_3_ are the applied loads, and their order of magnitudes are: *P*_3_ > *P*_2_ > *P*_1_.

Akbarzadeh and Khonsari [27] performed different loading sequence experiments on steel-on-steel and steel-on-brass tribo-pairs using a pin-on-disk test setup. They performed eight sets of experiments with the same magnitudes of loading amplitude but with the reverse loading sequence. From their experimental results, they observed a substantial difference (2.5 times greater) in measured weight loss when the magnitude of the loads is the same but with the opposite loading sequence.

**Figure 13:**
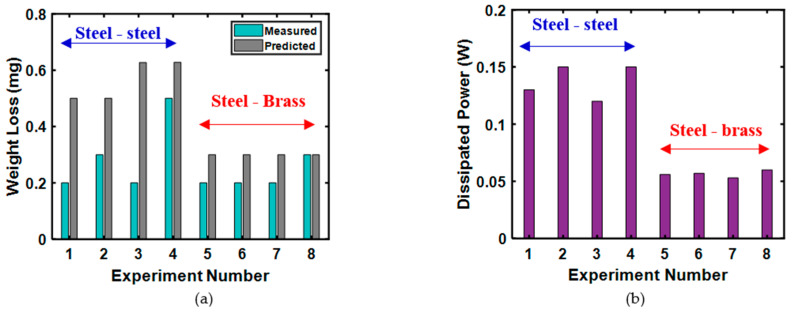
Comparison of weight loss between the measured and predicted weight loss using the Archard equation and determined dissipated power for steel-steel and steel-brass tribo-pairs (**a**) measured and predicted weight loss, (**b**) dissipated power [27].

Figure 13a presents the comparison of the measured and estimated weight loss using the Archard equation. From this Figure, it can be observed that the Archard equation cannot properly characterize the wearing of a tribo-pair experiencing variable loading. They also reported that the friction coefficient values changed with the loading sequence. The total value of dissipated power up to the point of tribo-pair failure (envisioned from the sudden increase in friction coefficient values) remained relatively constant (see Figure 13b).

Extending the work of Akbarzadeh and Khonsari [27], Lijesh and Khonsari [138] attempted to establish the efficacy of the degradation coefficient for the tribo-pair experiencing variable loading. They proposed a wear equation for variable operating conditions by correlating the wear of the tribo-pair to the load-dependent friction force and the contact temperature using the degradation coefficient (see Equation (27)).

(27) $$w_{vol_{l}}=B_{u,v}\overset{l}{\underset{l=1}{\sum }}\frac{P_{\mu }{}_{l}V_{l}}{T_{l}}t_{l}$$

where *B_u,v_* is the degradation coefficient during unidirectional sliding with a variable operating condition, $dw_{vol_{l}},\;P_{\mu }{}_{l}$, $V_{l}$ and $T_{l}$ are the wear volume, frictional force, sliding velocity, and the contact temperature at the *l^th^* load sequence, respectively.

**Figure 14:**
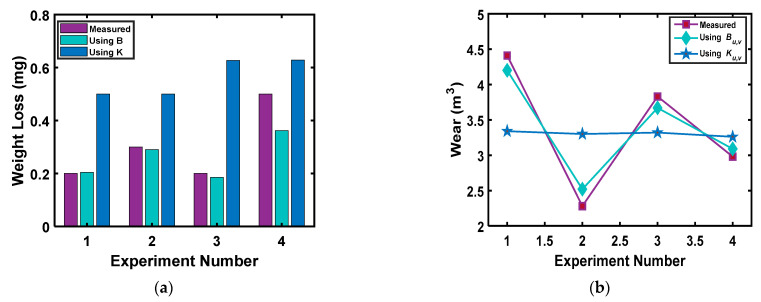
Comparison of weight loss/wear volume between the measured and predicted weight loss using Archard’s coefficient *K* and degradation coefficient *B* using the data from [138] (**a**) measured and predicted weight loss using *B* and *K,* (**b**) measured and predicted wear volume using *B_u,v_* and *K_u,v_*.

The effectiveness of Equation (27) is demonstrated using the experimental data from Akbarzadeh and Khonsari [27]. The comparison of the measured and estimated wear values using the Archard equation and DEG theorem is provided in Figure 14a. From this figure, it can be observed that the DEG theorem based on the thermodynamic framework can reliably predict the wear of a tribo-pair experiencing variable loading.

Lijesh and Khonsari [138] performed a series of experiments on a ball-on-disk test setup with a steel ball on brass specimens to further test the applicability of Equation (27). They considered four cases, in which the summation of the product of applied load and time duration of each equation was equal, i.e., $\overset{n}{\underset{l=1}{\sum }}P_{\mu }{}_{l}t_{l}=C$. The wear volume measured during the experiment, using constant values of the degradation coefficient *B_u,v_* (via Equation (28a)) and the Archard wear coefficient *K_u,v_* (via Equation (28b)) for all the four cases are plotted in Figure 14b.

This figure reveals that the wear volume changes with the loading sequence and the wear volume determined using the degradation coefficient *B_u,v_* can capture the effect of loading variations. In contrast, no variation in the wear volume can be observed using *K_u,v_* values.

(28a) $$B_{u,v}=w_{vol}\overset{l}{\underset{l=1}{\sum }}\frac{T_{l}}{F_{l}\mu_{l}V_{l}t_{l}}$$

(28b) $$K_{u,v}=w_{vol}\overset{l}{\underset{l=1}{\sum }}\frac{H}{P_{l}V_{l}t_{l}}\;$$

Therefore, it is concluded that a constant value of *B_u,v_* from the DEG theorem can predict the wear characteristics during a load sequence where *K_u,v_* fails.

Extending the work of Akbarzadeh and Khonsari [27], Fereidouni et al. [26] established that the cumulative power dissipation and entropy of the tribo-system experiencing loading variation stay comparatively constant and independent of the loading variation.

##### Loading and Sliding Speed Sequence Effect

Along with the variable loading conditions, Lijesh and Khonsari [138] demonstrated the efficacy of the thermodynamic approach in characterizing the variable sliding speeds and arbitrary combination of both load and sliding speed. The objective of their study was to determine the useful life of tribo-pair degrading due to adhesive wear under unlubricated conditions. To visualize the onset of the failure of the contacting pair, the authors coated the brass disk with black paint. When the ball contacted the brass surface after the coating was worn, the friction force rapidly increased and fluctuated erratically. Lijesh and Khonsari [139] performed experiments for variable operating conditions by varying (i) load (ii) sliding speed, and (iii) the arbitrary change of load and sliding speed. The experimental results were compared with the results obtained from the DEG theorem and by considering Miner’s constant as 1. Comparison to experimental results showed that the maximum error for Miner’s rule was determined to be 103%, while using the thermodynamic approach, the maximum error yielded ~10%.

#### 3.1.3. Bidirectional Sliding and Uniform Operating Condition

Most of the developed wear models are best suited for characterizing the wear of tribo-pairs undergoing a unidirectional sliding motion [140,141,142,143]. However, in practice, many machines experience a reciprocating or oscillatory motion that necessitates characterizing wear of a bidirectional sliding motion [92,144,145,146,147,148]. Determination of the useful life of components experiencing bidirectional motion is crucial to avoid catastrophic failure of the machine. For example, as a result of flow-induced vibration, 60 pressurized water reactors were reported to have leaked due to the bidirectional wear/corrosion of steam generator tubes [149]. Hwang et al. [150] also reported severe damage to steam generator tubes in Korean nuclear power plants. The San Onofre Nuclear Generating Station unit had to shut down due to the damage of the steam generator tube in 2012 [151].

**Figure 15:**
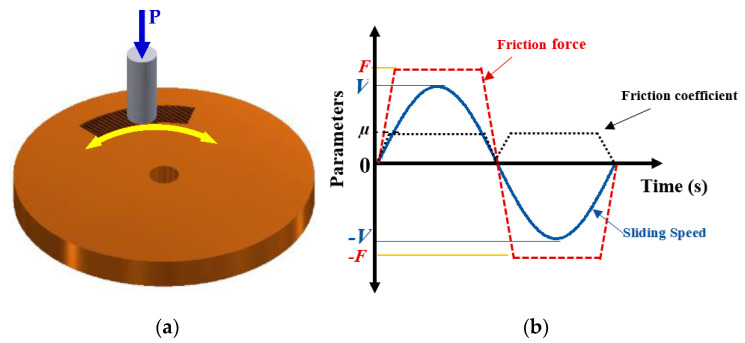
Bidirectional oscillation motion and parameters defining them (**a**) pin on disk with an oscillating motion, (**b**) friction force, friction coefficient, and sliding speed during oscillating motion.

During the bidirectional sliding motion (see Figure 15a), the magnitude of velocity *V* and friction force F varies between the negative and positive values, while the friction coefficient value *µ* remains positive and varies from zero to *µ* (see Figure 15b).

Research shows that applying the Archard equation to assess wear in a bidirectional sliding motion does not yield satisfactory results [147,148,152,153,154,155,156]. The reason is often attributed to factors such as the variation in friction force, wear mode, displacement amplitude, contact geometry, etc. In contrast, energetic and entropic characterization of wear in bidirectional motion confirms the experimentally observed linear relationship between the wear rate and the dissipated friction energy and entropy generation [143,144,145,156].

Lijesh and Khonsari [157] proposed a wear equation (Equation (20)) characterizing the wear of tribo-pairs in bidirectional motion using the degradation-entropy generation (DEG) theorem, which considers both the first and the second laws of thermodynamics along with degradation forces. It is worth mentioning that the term μVP in Equation (29) is the frictional energy dissipated during the sliding motion. This is equivalent to the expression provided by [5,152,158] but without considering the temperature. In other words, the energy dissipation expression used in references [5,152,158] is a subset of Equation (18). This suggests that the DEG theorem can be used to derive a generalized expression to characterize the wear of tribo-pair in a bidirectional sliding condition.

(29) $${\dot{w}}_{vol}=B_{b,u}\overset{n}{\underset{m=1}{\sum }}\frac{\mu_{m}V_{m}P_{m}}{T_{m}}\;$$

where the suffix *b* represents the bidirectional sliding condition. The variation of the magnitude of the velocity with time in the form of a sine or cosine waveform. Therefore, it is thought to be reasonable to replace the value of *V_m_* in Equation (29) with *V* Sin(*ωt*). Further, the value of *V* Sin(*ωt*) can be represented with the root mean square value of velocity, i.e., *V*/√2. The final simplified equation for determining the wear of a tribo-pair in bidirectional sliding motion is [156]:

(30) $${\dot{w}}_{vol}=\frac{B_{b,u}V}{1.414}\;\overset{n}{\underset{m=1}{\sum }}\frac{\mu_{m}P_{m}}{T_{m}}\;$$

To establish the efficacy of the degradation coefficient *B_b,u_* over the Archard wear coefficient *K_b,u_*, Lijesh and Khonsari [157] performed pin-on-disk experiments, with 304 stainless steel pins on brass disks, in bidirectional motion with variable operating conditions. Their experimental conditions covered a wide range of oscillatory sliding. For prediction purposes, the following equations for *B_b,u,_* and *K_b,u_* were derived.

(31a) $$B_{b,u}=1.414\frac{{\dot{w}}_{vol}}{V}\;\overset{n}{\underset{l=1}{\sum }}\frac{T_{l}}{\mu_{l}P_{l}}\;$$

(31b) $$K_{b,u}=1.414\frac{{\dot{w}}_{vol}}{V}H\;\overset{n}{\underset{l=1}{\sum }}\frac{1}{P_{l}}\;$$

Table 1 shows the reported degradation and wear coefficients for six different experimental conditions. The standard deviations between the six values of *K_b,u,_* and *B_b,u_* were 1.2 and 0.01, respectively. It is evident from the lower standard deviation of *B_b,u_* than *K_b,u_*, indicating that the variation of the degradation coefficient values among the experimental conditions is significantly lower than the wear coefficient.

**Figure 16:**
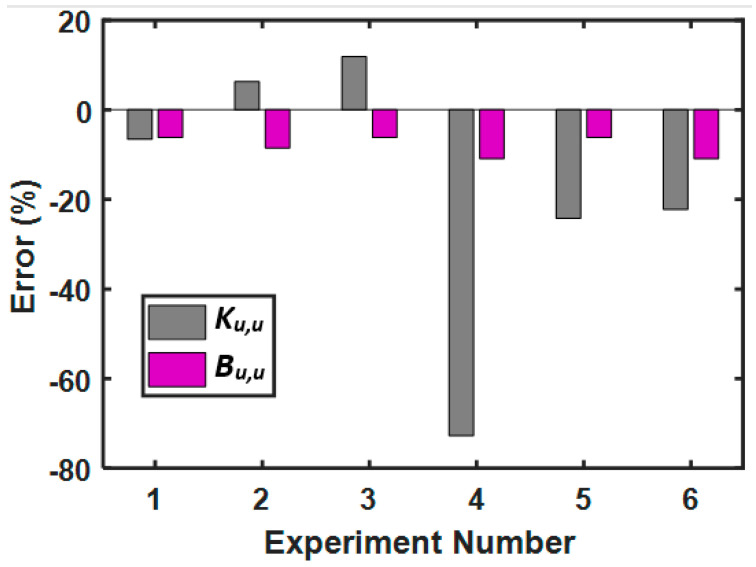
Error obtained for different coefficients between a bidirectional and unidirectional motion for different operating conditions [157].

To gain more insight, Lijesh and Khonsari [157] extended their study by examining the error obtained between the degradation and wear coefficients during bidirectional sliding with respect to values of *K_u,u,_* and *B_u,u_* obtained during unidirectional sliding. The values of *K_u,u_* and *B_u,u_* obtained from [138] for the considered tribo-pairs were 3.96 × 10^−4^ and 0.433 mm^3^K/J, respectively. The error for *K_b,u,_* and *B_b,u_* obtained with respect to *K_u,u,_* and *B_u,u_* of a unidirectional sliding is shown in Figure 16. Note that the percentage of error determined for the Archard coefficient is in the range of 11.87% to −72.73%, while for *B_b,u_* it is −6.2% to −10.9%. This reveals that the degradation coefficient is capable of providing a more realistic tool for determining the degradation of a tribo-pair operating in bidirectional sliding conditions.

**Figure 17:**
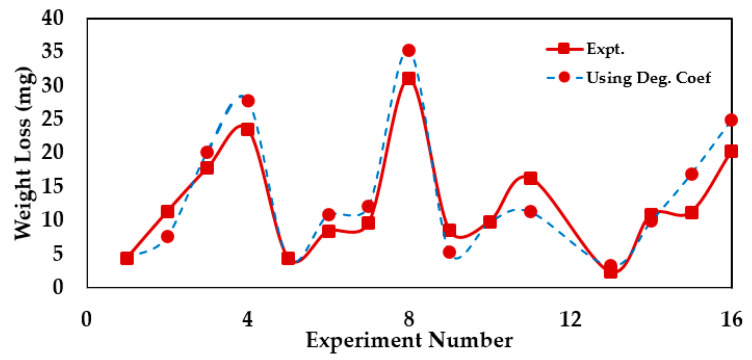
Comparison of wear data from experiment [159] and using *B_b,u_*. Adapted from [157].

Rajeev et al. [159] performed sixteen bidirectional sliding experiments on Al-Si-SiCp composites under different values of load, sliding distance, reciprocating velocity, experimental temperature, and friction coefficient. After completion of the experiment, the weight loss was measured. Since the degradation coefficient *B_b,f_* values for the considered tribo-pair were not provided in [159], the value of *B_b,u_* was determined for one of the experiments (first data in this case). From the calculated value of *B_b,u_* the values of wear for the other operating conditions were determined. The experimental wear and wear determined using *B_b,u_* are plotted in Figure 17.

**Figure 18:**
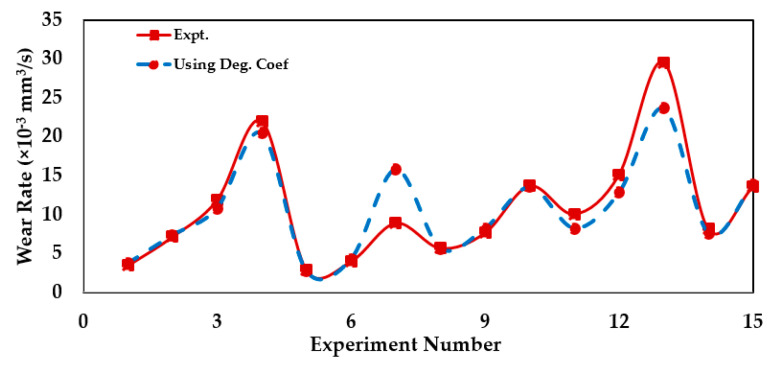
Comparison of wear rate values from experiment [97] and using *B_b,f_*. Adapted from [157].

Aghdam and Khonsari [97] performed fifteen bidirectional sliding experiments considering steel plate and pin for different stroke lengths, frequency, and load values. Having determined the degradation coefficient *B_b,u_* value for the first experimental condition, the wear for the other experimental conditions was determined. The wear rate determined using *B_b,u_* value, and the experimental wear values are plotted in Figure 18.

**Figure 19:**
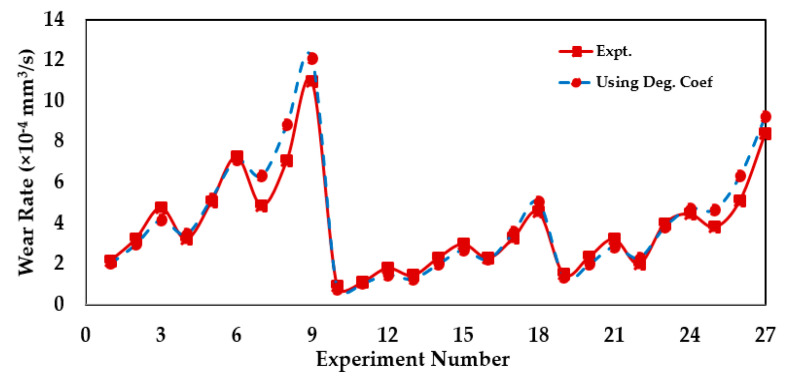
Comparison of wear rate values from experiment [97] and using *B_b,u_*. Adapted from [157].

A similar procedure is followed to characterize the wear behavior of a grease-lubricated bearing (commonly known as pin-bushing) operating in boundary-lubricated conditions and an oscillatory sliding motion. The application of such types of bearing is commonly employed in excavators, drag liners, etc. Aghdam and Khonsari [97] performed experiments under twenty-seven different operating conditions. Now, considering all the operating conditions, the wear rate values are determined using *B_b,u,_* and the values are plotted in Figure 19, along with the experimental wear rates.

The results of Figure 17, Figure 18 and Figure 19 suggest that wear values obtained using *B_b,u_* can accurately predict the degradation of the tribo-pair experiencing bidirectional sliding when the operating condition is uniform.

#### 3.1.4. Bidirectional Sliding and Variable Operating Conditions

In most practical applications, the operating conditions, such as load and speed, do not remain constant and vary with time. As discussed in the previous section, compared to a fixed operating condition, the degradation of the tribo-pair is more severe during a variable operating condition.

Lijesh and Khonsari [157] performed four sets of additional experiments to investigate the application of the degradation coefficient to characterize the degradation of the tribo-pair experiencing variable operating conditions under bidirectional oscillating motion. The operating conditions and the measured wear losses after completing the experiments are summarized in Table 2.

**Figure 20:**
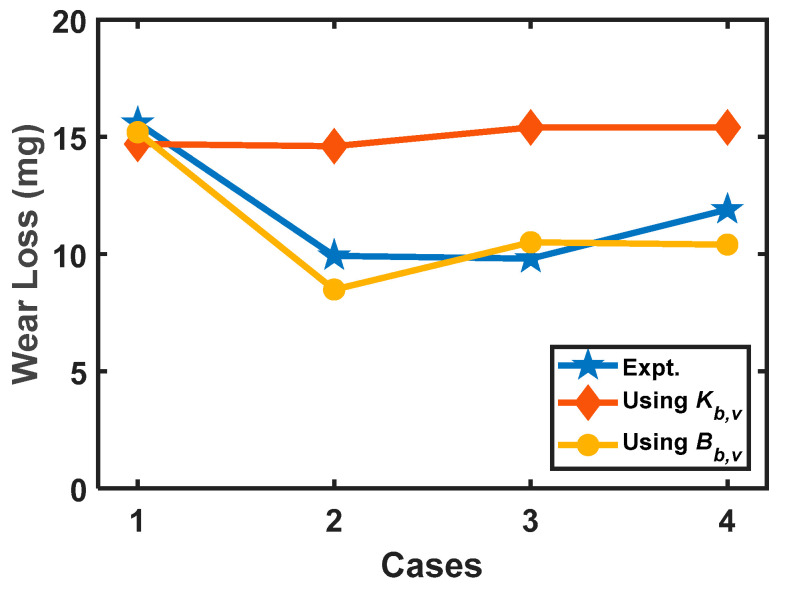
Comparison of wear loss from the experiment and determined using *K_b,v_* and *B_b,v f_* [157].

The wear loss is determined by applying the degradation coefficient via Equation (29). For comparison purposes, the wear coefficient was also calculated using Equation (30). The degradation coefficient *B_b,v_* and Archard’s wear coefficient *K_b,v_* for bidirectional sliding motion with variable operating conditions were determined using Equation (32a,b), respectively. The results for all four cases are plotted in Figure 20. From this figure, it can be observed that the wear loss measured from the experiment and determined using *B_b,v_* is approximately the same; however, the values of *K_b,v_* are observed to be constant, irrespective of the operating conditions. It can thus be concluded that the wear of a tribo-pair experiencing variable operating conditions with bidirectional sliding motion can be accurately characterized by degradation coefficient.

(32a) $$B_{b,v}=1.414\frac{{\dot{w}}_{vol}}{V}\;\overset{l}{\underset{k=1}{\sum }}\overset{n}{\underset{i=1}{\sum }}\frac{T_{i,k}}{\mu_{i,k}P_{i,k}}\;$$

(32b) $$K_{b,v}=1.414\frac{{\dot{w}}_{vol}}{V}H\;\overset{l}{\underset{k=1}{\sum }}\overset{n}{\underset{i=1}{\sum }}\frac{1}{P_{i,k}}\;$$

### 3.2. Running-In Wear

The term “running-in” is sometimes used in two different perspectives: (i) changes in friction and/or wear that occur in a tribo-system before it reaches a steady-state and (ii) the operational procedure used to condition the surfaces for achieving optimal friction or wear performance [160]. Blau [161] defined running-in as “the processes which occur prior to steady-state when two or more solid surfaces are brought together under load and moved relative to one another. These processes are usually accompanied by changes in nominal friction coefficient and/or rate of wear”. This view of running-in is universally accepted. The interested reader may refer to a detailed review of the state-of-the-art on this subject [162].

**Figure 21:**
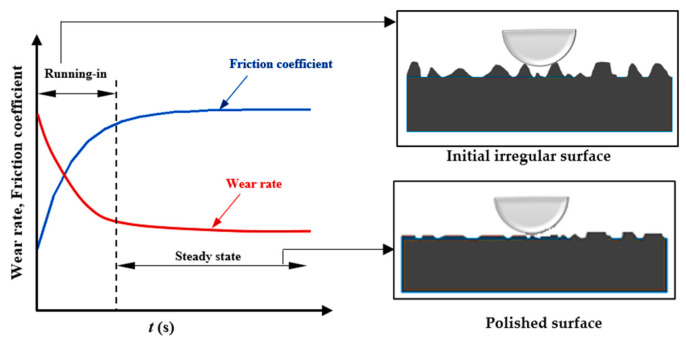
Transient wear and friction behavior during the wearing of the surface.

The initiation of the adhesive wear with running-in wear is due to the initial “surface irregularities” developed, owing to machining. During the rubbing process, the asperities undergo a polishing action. This results in a transient behavior wherein the tribological properties—e.g., wear rate, surface roughness, and friction coefficient—change in a nonlinear fashion until steady-state wear is reached [162]. This process is illustrated in Figure 21.

Characterizing and understanding the running-in process during adhesive wear is critical in determining the useful life of a tribo-pair, as the conditions of the sliding surfaces at the end of the running-in regime determine the steady-state performance. Research shows that an attempt to characterize the running-in wear using the steady-state Archard wear equation leads to a larger deviation in the value of *K* [163]. Reported deviations from measured values are as high as 52% [164] to 1000% [24].

The published report by Abbott and Firestone in 1933 [165] was probably the first study to address the running-in process associated with the change in surface geometry of the contacting surface. Blau [160] explored the concept of a running-in map and employed friction values to study the running-in process. He concluded that running-in is not only dependent on the material but also the entire tribo-system. Zhang et al. [166] analyzed the running-in behavior of 2014 Al matrix composite-steel tribo-pairs using the pin-on-disk setup and concluded that running-in behavior is dependent on the surface roughness and surface hardness. Blau [167] attributed the energy dissipation of the tribo-system being influenced by time-dependent and scale-dependent friction and wear phenomena occurring during the running-in process. Therefore, it is inferred that for characterizing the running-in wear, the model must consider the friction coefficient, hardness, roughness, and temperature.

In 1965, Queener et al. [168] provided an integrated wear model Equation (33) with two independent contributing components: transition and linear components (see Equation (33)).

(33) $$w_{vol}=K_{t}\left(1-exp\left(-Ex\right)\right)+K_{s}x$$

In Equation (32), the first term on the right-hand side, $K_{t}\left(1-exp\left(-Ax\right)\right)$, represents the transient wear of the surface irregularities associated with the pristine surface. The second term,$\;w_{s}=K_{s}x,$ represents the steady wear that dominates after the completion of the running-in wear. Here, the values of constant $K_{t}$ and $K_{s}$ depend on the applied load, sliding distance, surface roughness, and hardness of softer materials. Constants $K_{t}$ and E must be determined by performing experiments.

**Figure 22:**
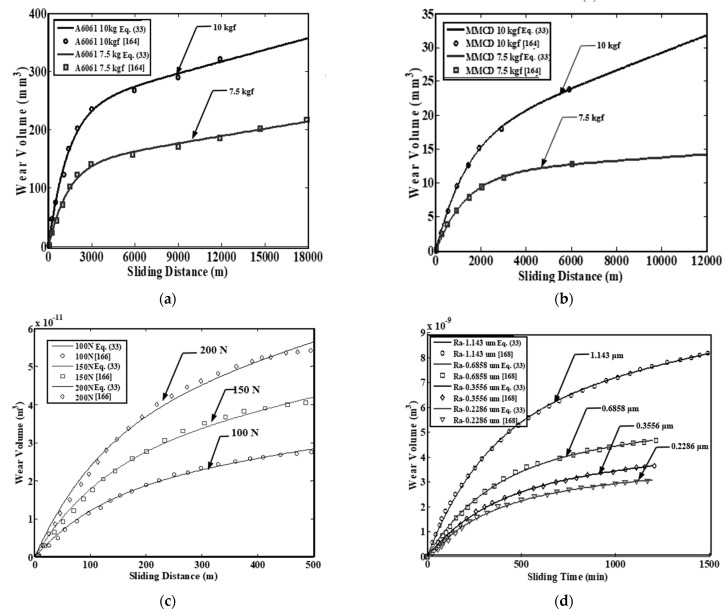
Experimental and predicted wear volume using Equation (33) from (**a**) Yang [164] (A6061) (1 kgf = 9.81 N) (**b**) Yang [164] (MMC-D) (1 kgf = 9.81 N) (**c**), Zhang et al. [166] (**d**) Queener et al. [168]. Data adapted from [173].

Several researchers [124,169,170,171,172,173] have employed integrated wear models to characterize the wear of tribo-pairs with both transient and steady-state wear. The experimental and wear volume values were determined using Equation (33) from [164,166,168] and shown in Figure 22. Equation (33) was found to provide good agreement with the experimental results. Further, employing Equation (33), Yang [164] observed 246% deviation in the predicted wear performance of metal matrix composites-D (MMC-D) via the integrated wear equation and using the Archard coefficient. It is worthwhile pointing out that the integrated wear model in Equation (33) did not consider the effect of surface roughness, hardness, and temperature.

Kumar et al. [174] characterized several pertinent parameters such as running wear, running-in period, and stead0y-state wear through a statistical approach. They provided polynomial equations for each parameter, considering load, temperature, and surface roughness as variables. The equation is provided in Equation (34).

(34) $$log_{e}Y=a_{0}+a_{1}log_{e}P+a_{2}log_{e}R_{q}+a_{3}log_{e}T$$

where *Y* value represents running wear, running-in period, and steady-state wear values.

To investigate the appropriateness of the linear relationship between transient wear with respect to initial surface roughness and running-in time, Mortazavi and Khonsari [175] conducted a dimensional analysis via the Buckingham Pi theorem. The parameters included in the analysis were the transient wear, initial surface roughness, and running-in time. The study yielded several new dimensionless groups: (i) nondimensional transient wear loss $w^{*}$ (Equation (35a)), (ii) nondimensional initial roughness R* (Equation (35b)), and (iii) nondimensional time t* (Equations (35c)).

(35a) $$w^{*}=w{\left(\frac{H}{N}\right)}^{1.5}\;$$

(35b) $$R^{*}=R{\left(\frac{H}{N}\right)}^{0.5}\;$$

(35c) $$t^{*}=tV{\left(\frac{H}{N}\right)}^{0.5}\;$$

**Figure 23:**
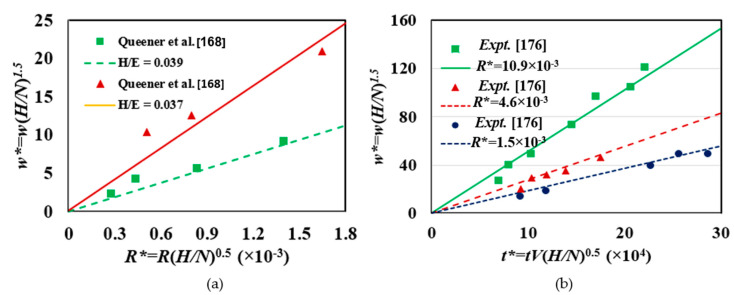
The linear relationship between transient wear, initial surface roughness, and running-in time [175]. (**a**) Relation of nondimensional transient wear loss and nondimensional initial roughness during running-in. (**b**) Relation of nondimensional transient wear loss and nondimensional time during running-in.

Mortazavi and Khonsari reported a linear relationship between (a) nondimensional transient wear loss and nondimensional initial roughness; and (b) nondimensional transient wear loss and nondimensional time. Their results are presented in Figure 23a,b, respectively.

Ghatrehsamani et al. [176] employed the continuum damage mechanics (CDM) method [177,178,179,180] to characterize the running-in wear. The CDM method relates wear coefficient and the number of cycles for the formation of a wear particle. Researchers Ghosh and Sadeghi [181], Albers and Reichert [182], Akbarzadeh and Khonsari [183,184], and Bosman et al. [185] characterized the running-in wear through a numerical approach. References [176,182,183,186] considered only roughness for characterizing the running-in wear while [184,185,186] included temperature as well in their model. However, the numerical methods are difficult to implement by the practitioner, for they require many additional material properties and much computational power to perform the necessary simulations.

**Figure 24:**
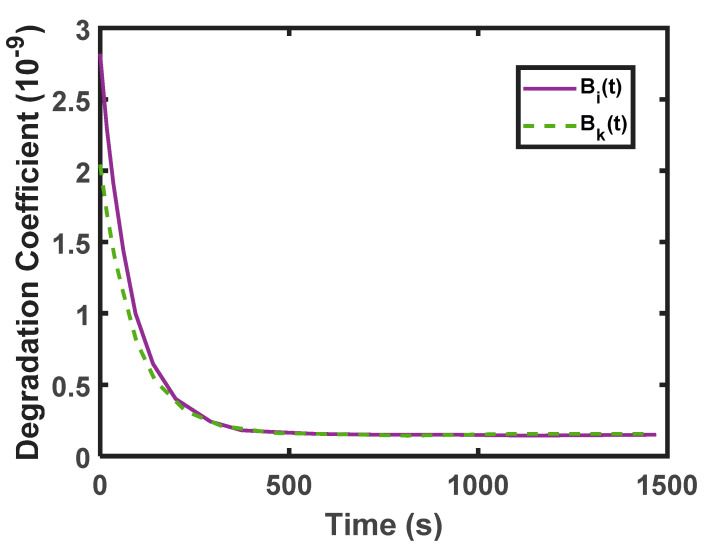
Estimated values of “*B_i_*(*t*)”, and “*B_k_*(*t*)”.

Using thermodynamic principles, Lijesh et al. [86] proposed a completely integrated analytical wear model to characterize the running-in wear. This model contains an integrated degradation coefficient, as shown in Equation (20). From Equation (16), degradation coefficient *B* is a function of the friction coefficient, hardness, and contact temperature. Lijesh et al. [86] performed experiments on a vertical pin-on-disk test setup, to prove the efficacy of the degradation coefficient $B_{i}\left(t\right)$ (in Equation (20)) compared to degradation coefficient $B_{k}\left(t\right)$ determined from the wear coefficient using Equation (16). The details of the test setup are given elsewhere [187,188]. The results obtained are plotted in Figure 24, and this Figure reveals that the transient wear predicted using the Archard coefficient deviated by ~27%, compared to the degradation coefficient. The deviation was due to the consideration of the time-dependent friction coefficient and contact temperature by the degradation coefficient. Finally, it was concluded that the degradation coefficient provides a realistic measure of wear during the running-in period.

In Table 3, we summarize the published work evaluating the characterization of degradation of tribo-pairs through thermodynamic principles.

**Figure 25:**
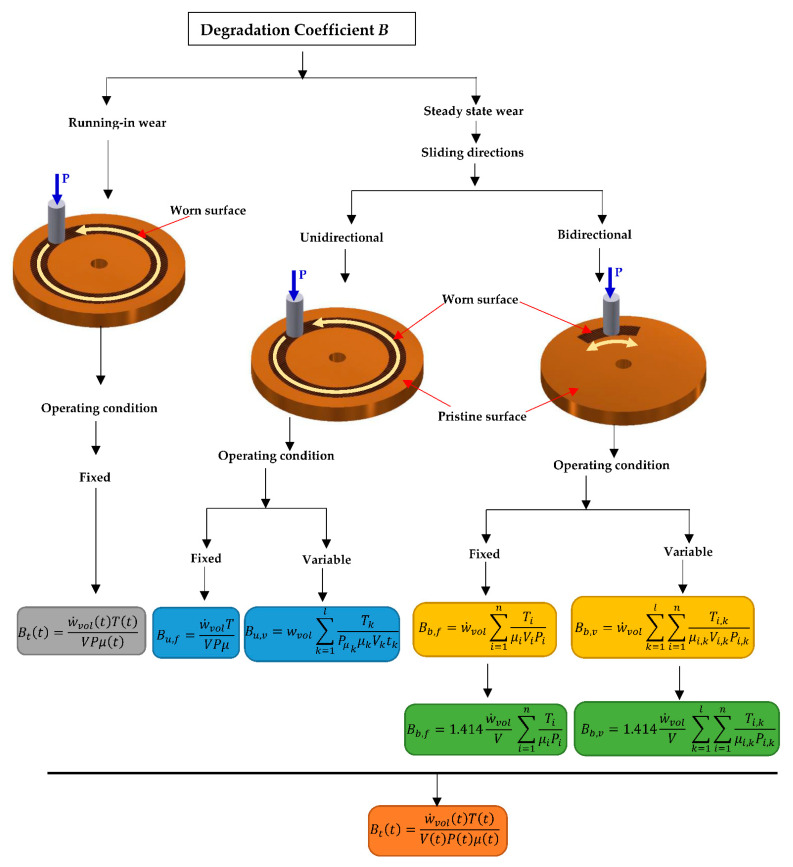
The consolidated equation for degradation coefficients for adhesive wear.

An illustrated summary of the equations for the degradation coefficient employed for different operating and sliding conditions is consolidated in Figure 25. In the present work, the focus of the review was on the adhesive type of wear under unlubricated conditions. However, research is needed to describe the path forward for analyzing more complex forms of wear involving multiple modes—e.g., combined adhesive and abrasive wear, mild to severe wear arising that leads to scuffing, galling, smearing, and seizing [189]—and wear in boundary- and mixed-lubricated regimes.

## 4. Conclusions

In the search for a unified wear equation, the authors carried out an extensive literature review on different wear models. The focus was on tribo-pair degradation due to the adhesive wear prevalent in different directions of sliding motion (uni- and bidirectional) and operating conditions (uniform and variable). It was inferred that the Archard equation (Equation (1)) provides satisfactory results for unidirectional and uniform operating conditions, while energy-based wear models (Equations (3)–(6)) were capable of characterizing degradation during bidirectional sliding and uniform operating conditions. The integrated wear model (Equation (33)) was capable of predicting the running-in wear. Other approaches such as numerical, dimensional analysis, CDM methods, etc., were also employed for characterizing degradation. However, these models/methods, require many additional material properties and considerable time and effort to simulate.

The degradation-entropy generation (DEG) theorem, developed according to thermodynamic principles, can provide a unified wear model. The DEG theorem relates the degradation of the tribo-pair to the irreversible dissipative processes connected to the degradation mechanism through the degradation coefficient *B*. It was found that the Archard law and the energy-based models are corollaries of the DEG theorem, and the theorem can be applied to the systems operating far from equilibrium, such as running-in. To establish the findings, the results of many recent reports were reviewed from the perspective of both theoretical and experimental validation. It is shown that the DEG theorem is able to correlate the degradation due to wear to entropy generation appropriately for all operating and sliding conditions.

## Figures and Tables

**Table 1:** Determined wear volume and error using *K_b,u_* and *B_b,u_*. Adapted from [157].

Cases	Load (N)	Sliding Speed (m/s)	Expt. Wear Loss (mg)	*K_b,u_* × 10^−4^	*B_b,u_* mm^3^K/J	Error (%)	
						Using *K_b,u_*	Using *B_b,u_*
1	5	0.063	2.8	4.22	0.46	−6.57	−6.2
2	5	0.126	4.9	3.71	0.47	6.31	−8.5
3	5	0.188	6.5	3.49	0.46	11.87	−6.2
4	10	0.063	9	6.84	0.48	−72.73	−10.9
5	10	0.126	13	4.92	0.46	−24.24	−6.2
6	10	0.188	17.7	4.84	0.48	−22.22	−10.9

**Table 2:** Four cases considered for the demonstration and measured wear loss. Adapted from [157].

Cases	Applied Load (N)				Sliding Speed (m/s)				Operation Time (min)				Wear (mg)
1	5	10	5	10	0.063	0.126	0.126	0.188	30	30	15	15	15.5
2	10	5	10	5	0.063	0.063	0.126	0.188	15	15	30	30	9.9
3	5	5	10	10	0.063	0.188	0.063	0.188	30	15	30	15	9.5
4	10	10	5	5	0.188	0.063	0.188	0.063	15	30	15	30	12

**Table 3:** Summary of studies of degradation of tribo-pairs based on thermodynamic principles.

Year	Authors [Ref.]	Method	Remarks
1965	Matveevsky [99]	Mathematical model, Experimental	Introduced friction power intensity term to measure the intensity of frictional energy dissipated in the contact region during the relative motion. First to report the correlation between energy dissipation and wear.
1967	Prigogine [121]	Mathematical model	Established that the entropy generation defines the tribo-system degradation. He insisted on developing formulas for the entropy generation in terms of experimentally measurable quantities.
1970	Bikerman [104]	Mathematical model	Described the importance of studying the friction behavior of tribo-pairs from the thermodynamic framework.
1980–1984	Klamecki [105,106,107,108]	Mathematical model	Presented a detailed thermodynamics analysis for characterizing the degradation of the tribo-system from the thermodynamics framework, when operated at nearly equilibrium conditions.
1987	Zmitrowicz [103,109,110]	Mathematical model	Developed formulae for characterizing thermodynamics of contacting bodies experiencing third-body interaction by using continuum mechanics, rational thermodynamics, and existing experimental results.
1995	Mohrbachcr [91]	Experimental	Reported a linear relationship between the wear volume of TiN coatings and the cumulative friction energy dissipated for bidirectional sliding in fretting wear.
1995	Plint [90]	Mathematical model, Experimental	Introduced energy pulse quantity to measure the severity of the wearing process by estimating the energy dissipation per unit area during the sliding motion.
1996	Fouvry et al. [93]	Experimental	For bidirectional sliding experiments, they reported a linear correlation between wear volume and dissipated energy for TiN-alumina and high-speed steel (HSS)-alumina.
1997	Huq and Celis [92]	Experimental	Via ball-on-disk experiments, they concluded that the measured wear loss was proportional to the total dissipated energy during a unidirectional sliding if the coating prevailed.
1998	Kondepudi and Prigogine [122]	Mathematical model	Expressed thermodynamic quantities of a perturbating system as the variations about the equilibrium state. He stated that during equilibrium state, the first variation of entropy production about the equilibrium state vanishes. In other words, for a transient system, the values of *dU* and *dS* are not zero.
1998	Celis et al. [94]	Experiment	Reported linear relation between wear volume and dissipated energy for hard-coated steel and alumina balls for different RH values.
1999	Huq and Celis [95]	Mathematical model, Experimental	Established a correlation between cumulated dissipated energy and wear volume by performing wear experiments on different types of coating at different relative humidity conditions. Characterization of the degradation process of tribo-system behavior becomes complex when the interaction of the surroundings is considered
1999–2005	Abdel-Aal [101,113,114,115,116,117,118,119]	Mathematical model, Experimental	Explored the correlation between the wear of contacting surfaces to the thermal properties of the materials from a thermo-mechanical perspective. He concluded that the wear volume is dependent on the amount of energy dissipated.
2000	Dai et al. [120]	Mathematical model, Experimental	For fretting wear system, the entropy reaches maximum and entropy generation concludes when operated near equilibrium. At equilibrium, the entropy flow and entropy production were equated and solved for wear by considering it as a mass flux component of entropy flow.
2000	Doelling et al. [17]	Mathematical model, Experimental	First time to establish the correlation between entropy flow and degradation of the component due to wear. They demonstrated that Archard’s equation is a thermodynamic consequence, and it is subsumed in their proposed wear−entropy relationship.
2001	Fouvry and Kapsa [154]	Experimental	Archard law failed to characterize the degradation of tribo-pair when the friction coefficient is not constant, while the energy dissipation approach will provide a stable quantification.
2002	Huq and Celis [5]	Experimental	Wear of materials in bidirectional sliding contacts was considered as resulting from an energy dissipation due to friction between the contacting first bodies.
2003	Fouvry et al. [152]	Experimental	This energy wear approach is applied to analyze hard-coating wear mechanisms focusing on abrasion and oxidation phenomena.
2006	Brahmeshwarkar [83]	Experimental	Experimentally verified correlation between wear and entropy flow in a tribo-pair operating in a unidirectional sliding motion with uniform/fixed operating conditions.
2008	Bryant et al. [84]	Mathematical model	Proposed a generalized theorem to characterize the irreversible degradation of a steady-state wearing system under relative motion. They called it the degradation-entropy generation (DEG) theorem. This theorem relates entropy generation to irreversible degradation via generalized thermodynamic forces X and degradation forces Y.
2008	Bryant and Khonsari [126]	Mathematical model, Experimental	Provided correlations for dry sliding wear and entropy flow through a degradation coefficient
2010	Amiri and Khonsari [33]	Review	Presented detailed review on the perspective of using the thermodynamic principles for contacting pairs.
2010	Bryant [46]	Review	Presented unification of different wear processes by considering the dissipative processes associated with the sliding interfaces.
2010	Beheshti and Khonsari [85]	Mathematical model, Experimental	Concluded that degradation of a tribo-pair is the direct consequence of an irreversible thermodynamic process involving friction and temperature.
2011	Aghdam and Khonsari [123]	Experimental	Performed experimental validation of the thermodynamic principle. They established that the degradation of tribo-pair is proportional to power and entropy generation.
2012	Amiri et al. [127]	Mathematical model, Experimental	Demonstrated the formulae for degradation coefficient can be obtained by using Buckingham’s dimensional analysis. Validated their findings with experimental results.
2013	Aghdam, and Khonsari [97]	Mathematical model, Experimental	From the correlation of average wear rate and the average power dissipation, they proposed power dissipation−wear rate factor, using which, wear rate is determined.
2016	Akbarzadeh and Khonsari [27]	Mathematical model, Experimental	Characterized the wear behavior of a tribo-pair under variable operating conditions by considering the Miner’s rule. For different operating conditions, the values of dissipated power for the failure of tribo-pair remained relatively constant.
2018	Lijesh et al. [86]	Mathematical model, Experimental	The applicability of the DEG for a transient wear condition such as the running-in condition was investigated.
2018	Lijesh and Khonsari [138]	Mathematical model, Experimental	Attempted to establish the efficacy of the degradation coefficient using DEG theorem for the tribo-pair experiencing variable loading.
2018	Lijesh and Khonsari [139]	Mathematical model, Experimental	Demonstrated the efficacy of the thermodynamic approach in characterizing the variable sliding speeds and arbitrary combination of both load and sliding speed.
2019	Lijesh and Khonsari [157]	Mathematical model, Experimental	Proposed a wear equation characterizing the wear of tribo-pairs in bidirectional motion with variable operating conditions using the degradation-entropy generation (DEG) theorem, which considers both first and second laws of thermodynamics, along with degradation forces. Validated with experimental results from [150,151].
2019	Fereidoun et al. [26]	Mathematical model, Experimental	Performed several experiments by varying loading sequence conditions and established that the cumulative power dissipation and entropy remain relatively constant and independent of the loading sequence.
2021	Khonsari et al. [162]	Review	A detailed review of the state-of-the-art on running-in wear is provided, along with wear from the perspective of thermodynamic principles.

## Data Availability

No supporting data is required.

## Nomenclature

*A*	The real area of contact (m^2^)
*A_p_*	Area of pin (m^2^)
*A_d_*	Area of the disk (m^2^)
*B*	Degradation coefficient (m^3^K/J)
*c*	Specific heat (J/kg/K)
EP	Energy pulse (Kg/m)
$E_{d}$	Energy dissipation (J)
$F_{t}$	Tangential force (N)
K	Wear coefficient
H	Hardness (Pa)
$\dot{m}$	The mass flow rate of wear particle (kg/s)
*P*	Applied load (N)
*p*	Degradation process
*p_c_*	Specific contact pressure (kg/m^2^)
*Q*	Heat flow (J)
ΔQ	Heat input (J)
$Q_{F}$	Specific power of friction (Kg/m/s)
$S_{e}$	Entropy flow (J/K)
$S_{g}$	Entropy generation (J/K)
*dS*	Change in entropy (J/K)
$d_{e}S$	Reversible change from entropy flow internally (J/K)
$d_{g}S$	Irreversible change from entropy generation internally (J/K)
*T*	Temperature (K)
*T_hc_*	The thickness of the control volume (m^3^)
*T_flash_*	Flash temperature (K)
*T_bulk_*	Bulk temperature (K)
*t*	Time (s)
*V*	Sliding velocity (m/s)
${\dot{w}}_{s}$	Steady-state wear volume rate (m^3^/s)
${\dot{w}}_{0}$	Initial wear volume rate (m^3^/s)
$w_{t}$	Transient wear volume (m^3^)
$w_{vol}$	Wear volume (m^3^)
${\dot{w}}_{vol}$	Wear rate volume (m^3^)
x	Sliding distance (m)
α	Heat partitioning factor
$\dot{\gamma }$	Entropy generation rate per volume (J/m^3^/K/s)
κ	Thermal conductivity (J/(smK))
*μ*	Friction coefficient
$\mu_{s}$	Static friction coefficient
$\mu_{0}$	Starting friction coefficient
*ρ*	The density of the material (kg/m^3^)
$\tau_{\mu }$	The time constant for transient behavior of friction coefficient (s)
$\tau_{w}$	The time constant for transient behavior of wear rate (s)
ζ	Time-dependent phenomenological variable
**Subscripts**	
*p*	Pin
*d*	Disk
*n*	Time interval
*J*	Dissipative processes

## Appendix A

### Appendix A.1. Different Wear Equations for Adhesive-Type Wear

**Table A1:** Adhesive wear equations.

Sl. No.	Ref.	Wear Equations
1	Holm [64]	$w_{v}=K_{ad}\frac{Px}{H}\;$ *K_ad_*—Archard wear coefficient, *P*—applied load, *x*—sliding distance, *H*—hardness of the softer material.
2	Mishina, and Alan [73]	$w_{v}=\frac{1}{3}\left(\frac{n}{\lambda }\right){\left(\frac{b}{a}\right)}^{3}\left(\frac{Px}{p_{m}}\right)$ *N*—Number of wear elements generated in the real contact area, *λ*—dependent on the chemical properties, *a*—junction radius, *b*—radius of wear element, *P*—applied load, *x*—sliding distance, *p_m_*—yield stress of softer material.
3	Finkin [74]	Wear proportional to number of interactions $w_{v}=N\left(\frac{x}{l_{m}}\right)P_{\infty }\left(1-e^{-\alpha l_{m}}\right){\bar{w}}_{v}$ Wear proportional to the area of asperity interaction $w_{v}=2N\left(\frac{x}{l_{m}}\right)l_{m}{\left(\frac{{\bar{A}}_{p}}{\pi }\right)}^{0.5}P_{\infty }\left(1-e^{-\alpha l_{m}}\right){\bar{w}}_{v}$ Wear proportional to the volume of slid interaction $w_{v}=N\left(\frac{x}{l_{m}}\right)A_{p}l_{m}P_{\infty }\left(1-e^{-\alpha l_{m}}\right){\bar{w}}_{v}$ *N*—number of plastic contacts, *x*—sliding distance, *l_m_*—mean asperity interaction length, ${\bar{w}}_{v}$—mean volume of individual wear particles, $P_{\infty }$—asymptotic value of probability of wear particle formation, α—inverse of some characteristic process.
4	Zhou et al. [79]	$w_{v}={\left(1-\gamma \mu^{2}\right)}^{0.5}\left(K_{e}A_{re}+K_{p}A_{rp}\right)x$ $A_{re}=\frac{D}{2-D}\left(S_{L}-S_{L}^{D/2}S_{c}^{\left(2-D\right)/2}\right)$ $A_{rp}=\frac{D}{2-D}S_{L}^{D/2}S_{c}^{\left(2-D\right)/2}$ *γ*—experimentally determined constant which relates to material hardening, lubricant, material interfacing, ploughing etc. *µ*—friction coefficient, *S_L_* is the area of the largest island, *S_c_*—critical area that distinguishes the elastic and plastic regimes, *D*—fractal parameter, *x*—sliding distance, *K_e_* and *K_p_* are the elastic and plastic wear coefficients respectively.
5	Yin, and Komvopoulos [81]	$w_{v}=\left(\frac{a^{\prime }-\pi \delta^{2}}{2}+\pi R\delta \right)dh-\pi \left(R-\delta \right)dh^{3}-\frac{\pi }{3}dh^{3}$ *a*′—truncated contact area or large-base area of spherical segment, *δ*—height of a spherical cap, *R*—equivalent radius of curvature of spherical asperity, *h*—global interference.
6	Nosonovsky, and Bhushan [82]	$w_{v}=K\mu PV$ *V* is the sliding speed.
7	Brahmeshwarkar [83]	${\dot{w}}_{v}=\frac{KT}{\mu H}\dot{S}$ $\dot{S}=\frac{A\frac{k_{s}\frac{T_{i}-T_{ii}}{d}}{1-\eta }}{T}$ $\eta =\frac{{\left(C_{p2}k_{2}\rho_{2}\right)}^{0.5}}{{\left(C_{p2}k_{2}\rho_{2}\right)}^{0.5}+{\left(C_{p1}k_{1}\rho_{1}\right)}^{0.5}}$ where *K* is the wear coefficient, *T* is the contact temperature, *µ* is the friction coefficient, $\dot{S}$ is the rate of entropy flow, *A* is the contact area, $T_{i}$ and $T_{ii}$ are the temperatures measured by the thermocouples at two locations separated by distance *d* along the direction perpendicular to sliding, *η* is the partition factor, *C_p_*, *k* and *ρ* are the specific heat capacity at constant pressure, thermal conductivity, and density of the material 1 and 2, respectively.
8	Bryant et al. [84]	$w_{v}=B_{ad}\frac{\mu Px}{T}$ *B_ad_*—degradation coefficient during adhesive wear, *µ*—friction coefficient, *P*—applied load, *x*—sliding distance, *T*—contact temperature.
9	Feinberg [87]	$w_{v}=\frac{\mu PS_{o}\;}{m{\bar{\sigma }}_{o}}\left(\varepsilon_{o}-\frac{\alpha \delta }{2}\right)\;\;\;\;\;\;\;\;\;\;\;\;\;\;\;\;\mathrm{if}\;\;\delta <x_{c}$ $w_{v}=\frac{\mu PS_{o}\;}{m{\bar{\sigma }}_{o}}\left(\varepsilon_{o}\frac{x_{c}}{\delta }-\frac{\alpha x_{c}^{2}}{\delta }+\frac{\varepsilon_{1}\delta^{-\beta }}{1-\beta }\left(1-{\left(\frac{x_{c}}{\delta }\right)}^{1-\beta }\right)\right)\;\;\;\mathrm{if}\;\;\delta >x_{c}$ *µ*—friction coefficient, *P*—normal load, *S_o_*—distance slid to create *n* wear sheets, *m*—constant, ${\bar{\sigma }}_{o}$ is the equivalent stress, δ—thickness of wear sheet.
10	Queener et al. [168]	$w_{v}=\beta \left[1-exp\left(-\emptyset x\right)\right]+Kx$ *K* is the steady wear coefficient, βis a parameter related to the surface roughness, and ∅ is the variable related to the load, speed, materials, and the environmental conditions.
11	Yang [169]	$w_{v}=K_{t}\left[1-exp\left(-Ax\right)\right]+K\frac{Px}{3H}$ The steady-state wear coefficients can be predicted by selecting *x* such that the value of $F_{A}=\left[1-exp\left(-Ax\right)\right]$ is slightly lower than unity. *K_t_*_,_ *A,* and *K* are constant.
12	Pawlus [124]	${\dot{w}}_{v}=\left({\dot{w}}_{t}-{\dot{w}}_{s}\;\right)exp\left(-\tau t\right)+{\dot{w}}_{s}$ ${\dot{w}}_{v},\;{\dot{w}}_{t}\;and\;{\dot{w}}_{s}$ are the total, transient and steady-state volume wear rate. τ is a constant to be determined.
13	Lijesh, and Khonsari [171]	${\dot{w}}_{v}\left(t\right)={\dot{w}}_{s}1+\left(\frac{{\dot{w}}_{0}}{{\dot{w}}_{s}}-1\right)e^{\left(-\frac{t}{\tau }\right)}$ $\tau =t\left[{\dot{w}}_{0}-0.632({\dot{w}}_{0}-{\dot{w}}_{s})\right]$ ${\dot{w}}_{0}\;=\left(\frac{{\dot{w}}_{\tau }}{x\tau -1}\right)\;\;\;\;\;\;\;$ $t_{s}=-\tau log\left[\frac{0.01}{\left(\frac{{\dot{w}}_{0}}{{\dot{w}}_{s}}-1\right)}\right]$ ${\dot{w}}_{s}=K_{R_{a}}R_{a}V\left[\left(\frac{E}{H}\right){\left(\frac{P}{H}\right)}^{0.5}\right]$ $K_{R_{a}}=\frac{w_{v}}{R_{a}x}\left[\left(\frac{H}{E}\right){\left(\frac{H}{P}\right)}^{0.5}\right]$ ${\dot{w}}_{0}$ wear rate at *t* = 0, *K_Ra_* is the modified wear coefficient with the inclusion of roughness, *R_a_* is the initial roughness of the surface, *E* is the Young’s modulus,
		$w_{v}=\beta +\alpha_{h}E_{d\;\;}$ *E_d_* is the dissipated energy, *β* and $\alpha_{h}$ are coefficients of the linear fit and $\alpha_{h}$ is called as energy density wear coefficient.
		$\delta =\alpha_{h}\sum E_{dh_{0}\;\;}$ $\begin{array}{c} \mathrm{when}\;e<1,\;\;\;\;E_{dh_{0}}=2q_{0}a\left(e{\left(1-e^{2}\right)}^{0.5}+arc\;sin\left(e\right)\right)\\ \mathrm{when}\;e>1,\;\;\;\;E_{dh_{0}}=q_{0}a\pi \; \end{array}\;\;\;\}$ $q_{0}=\frac{3P\mu }{2\pi a^{2}}$ *E_dho_* accumulated energy density, *e* sliding ratio, *δ_g_* is the stroke length, *a* is the contact radius,
		$w_{v}=\frac{A}{f_{0}\rho_{0}}E_{d\;\;}$ *A* is the area of contact, $f_{0}$ is the mass fraction of the oxide film and $\rho_{0}$ is the average density of the oxide formed at the real area of contact.

### Appendix A.2. Thermodynamic Force, Degradation Force and Thermodynamic Flow

The process of degradation process *p* can consist of *i* = 1, 2…*n* dissipative processes and these processes depend on a time-dependent phenomenological variable $\zeta_{i}^{j}\left(t\right)$, *j* = 1, 2,…*m* and produce an irreversible entropy $S_{g}$ characterized by the same set of phenomenological variable $\zeta_{i}^{j}\left(t\right)$. The accumulated effect of dissipative processes during the degradation of tribo-pair is given by

(A1) $$w_{v}=w_{v}\;\left[p_{i}\left(\zeta_{i}^{j}\right)\right]=\overset{n}{\underset{i=1}{\sum }}w_{v_{i}}\;$$

Now, according to the second law of thermodynamics, the degradation process generates a total irreversible entropy of

(A2) $$S_{g}=S_{g}\left\{p_{i}\left(\zeta_{i}^{j}\right)\right\}=\overset{n}{\underset{i=1}{\sum }}S_{g}{}_{i}$$

The rate of degradation ${\dot{w}}_{v}$ as well as entropy generation ${\dot{S}}_{g}$ can be represented as Equations (A1) and (A2), respectively.

(A3) $${\dot{w}}_{v}=\frac{dw_{v}}{dt}=\overset{n}{\underset{i=1}{\sum }}\overset{m}{\underset{j=1}{\sum }}\left(\frac{\partial w_{v}}{\partial p_{i}}\frac{\partial p_{i}}{\partial \zeta_{i}^{j}}\right)\frac{\partial \zeta_{i}^{j}}{\partial t}=\overset{n}{\underset{i=1}{\sum }}\overset{m}{\underset{j=1}{\sum }}Y_{i}^{j}J_{i}^{j}$$

(A4) $${\dot{S}}_{g}=\frac{dS_{g}}{dt}=\overset{n}{\underset{i=1}{\sum }}\overset{m}{\underset{j=1}{\sum }}\left(\frac{\partial S_{g}}{\partial p_{i}}\frac{\partial p_{i}}{\partial \zeta_{i}^{j}}\right)\frac{\partial \zeta_{i}^{j}}{\partial t}=\overset{n}{\underset{i=1}{\sum }}\overset{m}{\underset{j=1}{\sum }}X_{i}^{j}J_{i}^{j}$$

where $Y_{i}^{j}=\frac{\partial w_{v_{ab}}}{\partial p_{i}}\frac{\partial p_{i}}{\partial \zeta_{i}^{j}}$, $X_{i}^{j}=\frac{\partial S_{g}}{\partial p_{i}}\frac{\partial p_{i}}{\partial \zeta_{i}^{j}}$ and $J_{i}^{j}=\frac{\partial \zeta_{i}^{j}}{\partial t}$.

Bryant et al. [84] refer to *X* as the thermodynamic force, *Y* as the degradation force, and *J* as thermodynamic flow. It is worth observing from Equations (A3) and (A4) that both equations include the thermodynamic flow term and now, rearranging these equations leads to the definition of an important parameter termed degradation coefficient *B*, defined as a ratio between the degradation and thermodynamic forces.

(A5) $$\;B_{i}=\frac{Y_{i}^{j}}{X_{i}^{j}}$$

From Equations (A3)–(A5)

(A6) $${\dot{w}}_{v_{i}}=\overset{m}{\underset{j=1}{\sum }}\left(\frac{\partial w_{v}}{\partial p_{i}}\frac{\partial p_{i}}{\partial \zeta_{i}^{j}}\right)\frac{\partial \zeta_{i}^{j}}{\partial t}=\overset{m}{\underset{j=1}{\sum }}\frac{\left(\frac{\partial w_{v}}{\partial p_{i}}\frac{\partial S_{g}}{\partial p_{i}}\right)}{\left(\frac{\partial S_{g}}{\partial p_{i}}\frac{\partial p_{i}}{\partial \zeta_{i}^{j}}\right)}\frac{\partial \zeta_{i}^{j}}{\partial t}=\overset{m}{\underset{j=1}{\sum }}B_{i}X_{i}^{j}J_{i}^{j}=B_{i}{\dot{S}}_{g}{}_{i}$$
